# Changing plasma cytokine, chemokine and growth factor profiles upon differing malaria transmission intensities

**DOI:** 10.1186/s12936-019-3038-x

**Published:** 2019-12-05

**Authors:** Ruth Aguilar, Joseph J. Campo, Silvia Chicuecue, Pau Cisteró, Alba Català, Leopoldina Luis, Itziar Ubillos, Beatriz Galatas, Pedro Aide, Caterina Guinovart, Gemma Moncunill, Carlota Dobaño

**Affiliations:** 1 ISGlobal, Hospital Clínic, Universitat de Barcelona, Carrer Roselló 153 (CEK Building), 08036 Barcelona, Catalonia Spain; 2 Centro de Investigação em Saúde de Manhiça (CISM), Maputo, Mozambique

**Keywords:** *Plasmodium falciparum*, Cytokines, Chemokines, Growth factors, Malaria transmission intensity, Antibodies, Tolerance, Age, Immunity

## Abstract

**Background:**

Malaria epidemiological and immunological data suggest that parasite tolerance wanes in the absence of continuous exposure to the parasite, potentially enhancing pathogenesis. The expansion of control interventions and elimination campaigns raises the necessity to better understand the host factors leading to susceptibility or tolerance that are affected by rapid changes in malaria transmission intensity (MTI). Mediators of cellular immune responses are responsible for the symptoms and pathological alterations during disease and are expected to change rapidly upon malaria exposure or cessation.

**Methods:**

The plasma concentrations of 30 cytokine, chemokine and growth factors in individuals of all ages from a malaria endemic area of southern Mozambique were compared between 2 years of different MTI: 2010 (lower, n = 234) and 2013 (higher, n = 143). The effect of the year on the correlations between cytokines, chemokines and growth factors and IgGs to *Plasmodium falciparum* (markers of exposure) was explored. The effects of age, sex, neighbourhood and parasitaemia on analyte levels and their interactions with year were also assessed.

**Results:**

An inverse correlation of several cellular immune mediators with malarial antibodies in 2013, and a lack of correlation or even a positive correlation in 2010 were observed. Most cytokines, chemokines and growth factors, regardless of their immune function, had higher concentrations in 2010 compared with 2013 in *P. falciparum*-infected and uninfected subjects. Age and neighbourhood showed an effect on analyte concentrations.

**Conclusions:**

The results show a different regulation of the cellular immune response in 2010 vs 2013 which could be related to a loss of immune-tolerance after a decline in MTI in 2010 and previous years, and a rapid re-establishment of tolerance as a consequence of more continuous exposure as MTI began increasing in 2012. Cellular immune mediators warrant further investigation as possible surrogates of MTI-associated host susceptibility or tolerance.

## Background

The burden of malaria disease has experienced significant changes in endemic areas in the 21st century. Between 2000 and 2015, an expansion of malaria interventions helped to reduce malaria incidence by 37% globally, and by 42% in Africa. However, between 2015 and 2017 no significant progress was made, with an estimated 219 million cases and 435 000 related deaths in 2017 [1].

Geographical and temporal changes in malaria transmission intensity (MTI) can affect disease burden and *Plasmodium* parasite dynamics. Thus, the expansion of control interventions and elimination campaigns raises the necessity to better understand the host factors affected by rapid changes in MTI.

In endemic areas of Africa, naturally acquired immunity (NAI) to malaria is developed with age and exposure to *Plasmodium falciparum* infection. NAI is suggested to be comprised of two main components: (i) an anti-parasite component, resulting in control of parasite replication and parasite clearance, which takes years to be acquired and is never sterilizing [2, 3]; and (ii) an anti-disease component, consisting of the ability to tolerate parasites asymptomatically, which is acquired rapidly and can result in long periods without malaria symptoms in older individuals [4, 5].

Tolerance is a less understood phenomenon. From the immunological perspective, it is defined as any endogenous mechanism by which a potentially injurious immune response is prevented, suppressed, or shifted to a non-injurious response [6]. In malaria, such tolerance developed by the host is suggested to be multi-factorial, including: (i) the neutralization of parasite toxins and other virulence factors; (ii) immuno-regulatory processes that reduce the damage triggered by excessive immune responses of the host; and (iii) cellular and systemic adaptive responses that limit the deleterious effects associated with stress imposed by pathogens and/or host immunity [7].

Epidemiological and immunological data suggest that anti-parasite immunity and tolerance wane in the absence of continuous exposure to the parasite [8], and changes in MTI likely affect anti-malarial immunity. In fact, the geographical distribution of malaria prevalence, morbidity and mortality depends directly upon MTI. In low MTI settings, exposed people are at a higher risk of severe disease. In high MTI settings, severe disease is limited to naïve individuals (visitors, infants), young children, and pregnant women, while the rest of adults tolerate the presence of parasites [8].

Temporally, immune adults who migrate to non-endemic areas are at significant risk of contracting malaria upon return to an endemic area, even increased susceptibility to severe malaria [9]. Previous studies show that IgG responses to malaria-specific antigens are maintained to a large extent upon cessation of malaria exposure, suggesting a long-lasting anti-parasite immunity [10]; however, control of pro-inflammatory responses and tolerance to *P. falciparum* appeared to be reduced [11]. Thus, a rapid decrease in exposure to *P. falciparum* would result in a lesser development of NAI in children, and a possible partial loss of previous immunity and tolerance in the older population if exposure was very low or discontinued, which may enhance malaria pathogenesis.

Cytokines, chemokines and growth factors mediate cellular responses and are responsible for the symptoms and pathological alterations during disease. The outcome of infection depends on the regulation of pro-inflammatory and anti-inflammatory responses, leading to protection or immunopathology [12]. Thus, blood soluble mediators are expected to change rapidly upon malaria exposure or cessation, and to reflect changes in anti-disease immunity and tolerance associated with varying MTI. Some *P. falciparum*-specific cytokine responses have been associated with protection against clinical malaria, including interferon gamma (IFN-γ) [13–17], interleukin-10 (IL-10) and tumour necrosis factor (TNF) [18, 19]. Others, such as IL-6, have correlated with increased risk of clinical malaria [12, 20]. Serum cytokines like IL-5 and RANTES appear to be important in the pathogenesis of severe malaria [20–23].

In this study, changes in cellular immune mediator profiles in 2 years of different MTIs were assessed in individuals of all ages from a malaria endemic area of southern Mozambique. To address this, a comprehensive panel of 30 cytokines, chemokines and growth factors, several of them known to vary upon malaria infection and/or exposure [11, 24, 25], were measured in plasma samples collected in two cross-sectional surveys in 2010 and 2013 in the Manhiça District. Most analytes had higher concentrations in 2010 (lower MTI) compared to 2013 (higher MTI) in *P. falciparum* infected but also in uninfected subjects. This could be indicative of a loss of immune-tolerance after years of decline in MTI, and a re-establishment of the tolerance as a consequence of a more continuous exposure due to a rise in MTI starting before 2013. The possibility of cytokines as surrogates of loss of parasite tolerance upon changes in MTI deserves further investigation.

## Methods

### Study design, subjects and sample collection

This study was developed in the context of yearly cross-sectional surveys performed to monitor changes in malaria burden and MTI in the Manhiça District, Maputo Province, in southern Mozambique, which started in 2010 and are still on going. The characteristics of the study area have been described in detail elsewhere [26]. The climate is subtropical and transmission of *P. falciparum* malaria is perennial and of moderate intensity, with two different seasons, a warm and rainy season from November to April, and a cool and dry season the rest of the year [27]. The cross-sectionals were performed at the end of the peak of the transmission season (February–March) and lasted approximately 1 month. Data on monthly mean rain in the 5 months previous to the 2010 and 2013 surveys are shown in Additional file 1: Table S1 (CHIRPS: http://chg.geog.ucsb.edu/data/chirps/). The cumulative rainfall levels between the months of September 2009 and January 2010 was 310.76 mm, and doubled to 625.26 mm between the months of September 2012 and January 2013. Individuals of all ages were selected by random age-stratified sampling from the demographic surveillance system census and were invited to participate in the study. Weighted sampling was done according to the following age groups: Infants < 1 year; 1 ≤ 2 years; 2 ≤ 3 years; 3 ≤ 4 years; 4 ≤ 5 years; 5 ≤ 10 years; 10 ≤ 20 years; 20 ≤ 40 years; 40 ≤ 60 years; ≥ 60 years. Participants were recruited from six different neighbourhoods: Malavele, Manhiça and Maragra, historically considered of low MTI; and Ilha Josina, Palmeira and Taninga, of moderate MTI [28]. A standardized questionnaire was filled-in with basic demographic information, use of malaria control tools and socio-economic status. Axillary temperature was measured and registered in the questionnaire. A blood smear was collected to determine *Plasmodium* parasitaemia according to standard, quality-controlled procedures [29].

Haemoglobin was measured using HemoCue portable devices (Ängelholm, Sweden). A blood aliquot was collected in an EDTA microtainer and plasma separated by centrifugation and cryopreserved at − 80 °C for immunological analyses. Blood was also collected onto filter paper for IgG quantification by ELISA [30], and for parasitaemia quantification by real time quantitative PCR (qPCR), as described elsewhere [31]. Febrile infection was defined as the presence of asexual *P. falciparum* parasites in blood detected qPCR, together with fever or reported fever during the previous 24 h. Febrile infections with parasites also detected by microscopy were considered clinical malarias. Anemia was defined as haemoglobin < 11 g/dL. Participants presenting with parasitaemia, anaemia, fever or history of fever in the previous 24 h were treated according to standard procedures. All malaria infections were treated with the first-line anti-malarial treatment (Coartem), and anaemia cases received ferrous sulphate, according to national guidelines. Participants presenting signs/symptoms of severity were transferred to the Manhiça District Hospital.

Plasma samples analysed in this study were from 377 participants from the surveys performed in 2010 (N = 234, 96 infected and 138 non-infected) and 2013 (N = 143, 65 infected and 78 non-infected). Years 2010 and 2013 were defined as lower and higher MTI periods, respectively, based on the trends reported in Mozambique, with a decrease in malaria prevalence from 2007 to 2011, and an increase since 2012 [32, 33].

All plasmas from infected individuals (161 participants qPCR positive) and the plasmas from 216 non-infected participants (qPCR negative) were analysed separately to assess cytokine profiles in 2010 and 2013 during an infection and at baseline, respectively. The plasmas from non-infected individuals were randomly selected from the surveys, balancing between years and stratifying by specific age groups (1 ≤ 2, 2 ≤ 5, 5 ≤ 10, 20 ≤ 40 and ≥ 60), with the last two groups enriched to have a sample size powered to address older age effects.

### Cytokine, chemokine and growth factor multiplex bead array assay

The Cytokine Human Magnetic 30-Plex Panel from Life Technologies™ was used to measure the concentrations (pg/mL) of the following cytokines, chemokines and growth factors in plasma: epidermal growth factor (EGF), fibroblast growth factor (FGF), granulocyte colony-stimulating factor (G-CSF), granulocyte-macrophage colony-stimulating factor (GM-CSF), hepatocyte growth factor (HGF), vascular endothelial growth factor (VEGF), tumour necrosis factor (TNF), interferon (IFN)-α, IFN-γ, interleukin (IL)-1RA, IL-1β, IL-2, IL-2R, IL-4, IL-5, IL-6, IL-7, IL-8, IL-10, IL-12(p40/p70), IL-13, IL-15, IL-17, IFN-γ induced protein (IP-10), monocyte chemoattractant protein (MCP-1), monokine induced by IFN-γ (MIG), macrophage inflammatory protein (MIP)-1α, MIP-1β and regulated on activation normal T cell expressed and secreted (RANTES) and eotaxin. This panel has been used in previous studies showing several analytes varying upon malaria infection and/or exposure [11, 24, 25].

Twenty-five microlitres of all plasmas were tested in single replicates applying a modification of the manufacturer’s protocol that implies using half the volume of each reagent except for the washing buffer [34]; this modification was previously tested, showing no difference in assay performance compared to the original protocol. Each plate included 16 serial dilutions (two fold) of a standard sample provided by the vendor with known concentrations of each analyte, two blank controls and three positive controls of high, medium and low concentrations in duplicate prepared from a reference sample for quality assurance/quality control purposes. Samples from infected and non-infected individuals were assayed in two separate batches. In each batch, plates were balanced across the two cross-sectionals and age groups. Samples were acquired on a Luminex^®^ 100/200 instrument and analysed in xPONENT^®^ software 3.1. The concentration of each analyte was obtained by interpolating the median fluorescent intensity (MFI) (after blank MFI subtraction) to a 5-parameter logistic regression curve automatically calculated by xPONENT^®^ software. Any value below the lower limit of detection (mean of blanks + 2 standard deviations) was assigned half the expected concentration at the low limit of quantification for that analyte.

### Enzyme-linked immunosorbent assay

Antibody data obtained in a separate study of seroconversion rates performed in the context of the Manhiça cross-sectional surveys were analysed in relation to the cytokine data. Briefly, 2.5 mm disks of dried blood (≈ 1.5 µL of blood) were cut from filter paper spots and incubated overnight at room temperature (RT) with 150 mL of PBS/0.05% Tween 20/0.01% sodium azide (w/v) [35].

Reconstituted sera were stored at − 20 °C until use. The ELISA assay was performed as previously described [30]. High-binding 96-well titer plates (Immulon 4HBX, Thermo Scientific, Inc.) were coated with merozoite surface protein-1 (MSP-1_42_) (3D7 strain) or apical membrane protein-1 (AMA-1) (FVO strain) produced at the Walter Reed Army Institute of Research (MD, USA), at 0.5 mg/mL of carbonate buffer. PBS with 0.05% Tween (PBS-T) was used to wash plates between incubations. Plates were blocked with 1% skimmed milk powder (Sigma-Aldrich, Inc.) in PBS-T (blocking buffer) for 3 h at RT. Reconstituted antibodies were transferred to the ELISA plates (1/1000 and 1/2000 final dilution for MSP-1_42_- and AMA-1-coated plates, respectively) and incubated overnight at 4 °C. HRP-conjugated rabbit anti-human IgG (Dako/Agilent Technologies, Inc.) was applied at 1/5000 in PBS-T and incubated for 3 h at RT. OPD development substrate was applied and incubated in the dark at RT for 20–25 min, stopping development with the addition of 2 M H_2_SO_4_. Plates were read on Bio-Tek ELx 50 plate reader using KC Junior software package (version 1.10, Biotek Instruments Inc.). Normalized optical density (OD) was calculated as the mean background-adjusted OD divided by the mean of a hyperimmune plasma. The same standard dilution was used for normalizing each plate in the study. Test samples were assayed in duplicate and included in the analysis if the coefficient of variation was less than 50% for all values greater than 0.1 OD.

### Statistical analysis

Plasmas from malaria-infected and non-infected volunteers were analysed separately. The studied population was categorized into 6 age groups (≤ 2 years, > 2 ≤ 5 years, > 5 ≤ 10 years, > 10 ≤ 20 years, > 20 ≤ 60, and ≥ 60 years) according to commonly observed immunological patterns. Demographic continuous variables were analysed using the non-parametric Wilcoxon rank-sum test. Comparisons between groups for categorical variables were done using Fisher’s exact test. Concentrations of cytokines, chemokines, growth factors, parasite densities and IgG levels (OD) were log_10_ transformed for further analysis.

Comparison of cellular analyte concentrations between two or more groups was performed through Wilcoxon rank-sum test or Kruskall–wallis test, respectively. Comparisons of IgG levels between years were performed by Wilcoxon rank sum test with continuity correction for all age groups together or into each age category separately. Trends of IgG levels along age groups were assessed by Spearman correlations (p-trend). The correlations of IgG levels and analyte concentrations were assessed with univariable linear models, with IgG levels as independent variable, separate for each analyte. The effects of year, age group, neighbourhood, sex and parasite density (predictors) on analyte levels (outcome) were assessed through univariable and multivariable (adjusted models) linear regressions, separate for each analyte. Interaction tests were performed to determine if there was an interaction of age group, neighbourhood, sex, parasite density and antibody levels with year on the analyte levels. When interactions were statistically significant, a stratified analysis was performed.

All p-values were considered statistically significant when < 0.05. *P*-values were adjusted for multiple testing to control the false discovery rate using the Benjamini–Hochberg approach in each study endpoint separately (effect of year in infected subjects, effect of year in uninfected subjects, comparison of infected and uninfected subjects, effect of age, neighbourhood, sex,  parasitaemia and AMA-1 and MSP-1 IgG levels) except for the comparison of antibody levels between years into age categories for which the Holm method was used. In all endpoints evaluating cytokine associations of this exploratory study, the Benjamini–Hochberg approach was used because it has more power than family-wise-error rate methods, such as Bonferroni or Holm, and allows identifying as many significant associations as possible while incurring a relatively low proportion of false positives [36]. For the AMA-1 and MSP-1 antibody data a family-wise error rate method was used to be more conservative and because only two markers were analysed. All data collected were analysed using the R software version 3.2.4 (2016-03-10) [37]. The *ggplot2* package [38] was used to perform boxplot graphs and scatter plots. The *compare Groups* package was used for computing p-trend values [39]. The *fmsb* package [40] was used to draw the radar charts with the *radarchart* function. The *reshape* and *dyplr* packages were used for data manipulation purposes [41] and the *ReporteRs* package for exporting data tables in Microsoft Word documents [42].

## Results

### Malaria prevalence and antibody levels in the 2010 and 2013 surveys

A total of 981 and 980 individuals participated in the malaria cross-sectional surveys in 2010 and 2013, respectively. In 2010, 975 (99.5%) individuals were tested by microscopy and 970 (99%) by qPCR; while 944 (96.3%) and 808 (82.4%) individuals were tested by microscopy and qPCR, respectively, in 2013. Weighted prevalence of *P. falciparum* infection in 2010 and 2013 were 1.1% and 5.2% by microscopy, and 12.6% and 23.9% by qPCR, respectively; and the numbers of febrile infections were 15 and 43, being 3 and 18 clinical malarias, respectively (Table 1). When stratifying by neighbourhood, Palmeira, Ilha Josina and Taninga showed the highest increases in malaria prevalence (qPCR) from 2010 to 2013, with Palmeira and Ilha Josina presenting the highest increases in clinical cases.

**Table 1:** Weighted malaria prevalences and number of febrile infections in the study area and neighbourhoods, determined by microscopy and real time quantitative polymerase chain reaction (qPCR)

	Diagnostic	2010						2013					
		Individuals tested	Infected	Weighted prevalence of infection (%)	Lower 95% CI	Upper 95% CI	Febrile infections	Individuals tested	Infected	Weighted prevalence of infection (%)	Lower 95% CI	Upper 95% CI	Febrile infections
Whole study area^a^	Microscopy	975	13	1.11	0.56	2.21	3 (23%)	944	45	5.22	3.64	7.42	18 (40%)
	qPCR	970	106	12.59	10.09	15.6	15 (14%)	808	162	23.86	20.18	27.99	43 (26%)
Maragra	Microscopy	224	3	0.9	0.22	3.64	1 (33%)	206	5	3.9	1.57	9.35	4 (80%)
	qPCR	223	33	17.31	11.85	24.6	3 (9%)	171	23	18.8	12.19	27.86	5 (22%)
Manhiça	Microscopy	297	2	0.23	0.06	0.91	1 (50%)	362	12	3.21	1.51	6.68	2 (17%)
	qPCR	299	27	9.27	5.84	14.42	4 (15%)	298	45	17.37	12.34	23.89	6 (13%)
Malavele	Microscopy	56	1	2.55	0.36	16.13	1 (100%)	52	4	11.87	3.55	33.05	2 (50%)
	qPCR	55	7	13.75	5.68	29.67	2 (28%)	48	10	19.49	8.35	39.17	5 (50%)
Palmeira	Microscopy	206	3	1.11	0.27	4.46	0 (0%)	195	6	3.43	1.31	8.68	2 (33%)
	qPCR	205	15	8.9	4.84	15.8	1 (7%)	174	42	31.19	22.91	40.87	12 (28%)
Ilha Josina	Microscopy	124	3	4.2	1.11	14.65	0 (0%)	76	17	24.47	13.94	39.33	7 (41%)
	qPCR	120	21	23.95	14.73	36.47	4 (19%)	70	34	52	36.98	66.67	13 (38%)
Taninga	Microscopy	68	1	0.51	0.07	3.58	0 (0%)	52	1	0.5	0.07	3.56	1 (100%)
	qPCR	68	3	5.68	1.48	19.4	1 (33%)	46	8	22.24	10.09	42.16	2 (25%)

^a^11 individuals participated in both surveys

**Fig. 1:**
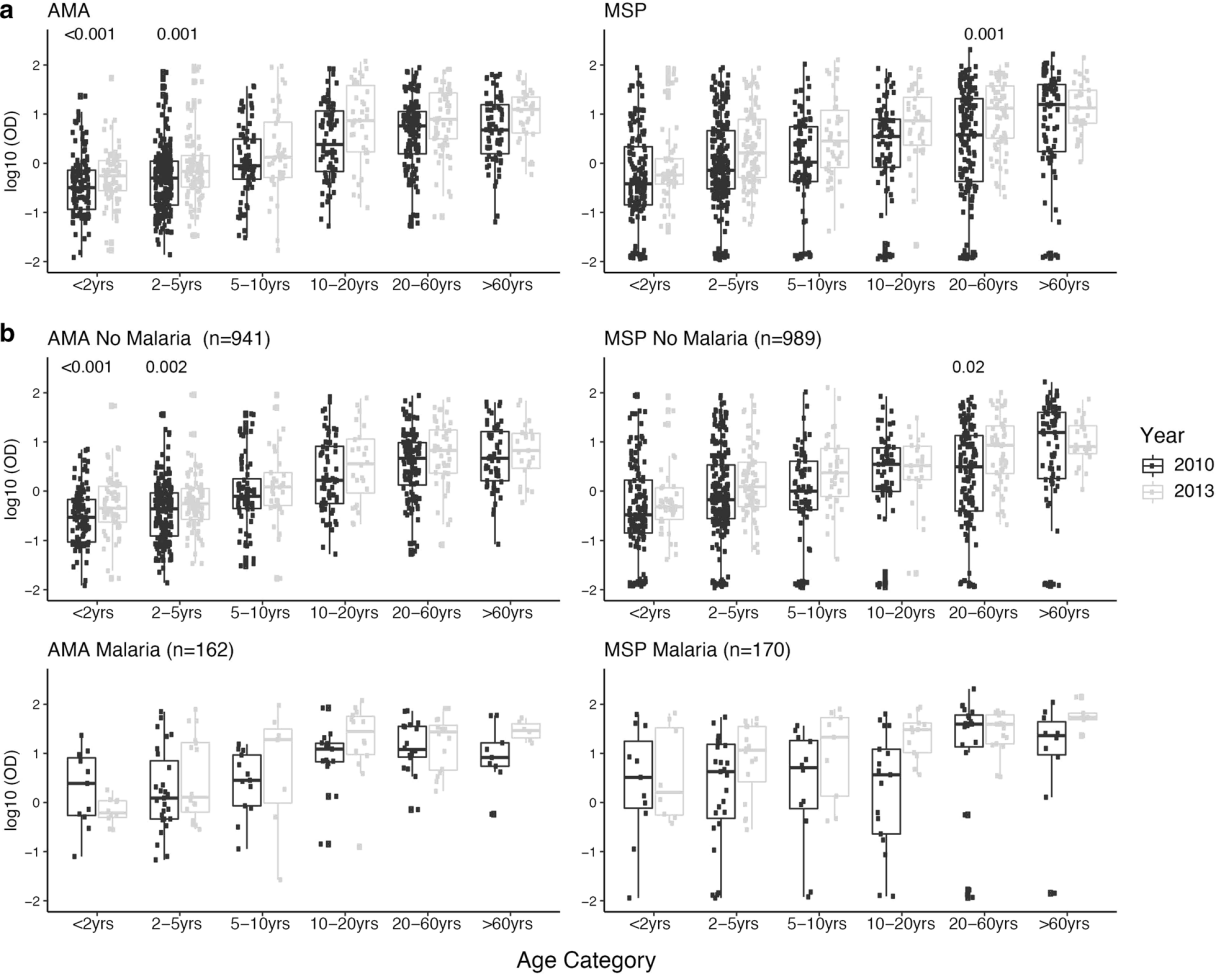
Differences in IgG levels against apical membrane protein-1 (AMA-1) and merozoite surface protein-1 (MSP-1_42_) between 2010 and 2013 in each of the age groups (**a**) and stratifying by *P. falciparum* infection detected by qPCR (**b**). Box plots representing the median and interquartile range of IgG levels (log_10_ OD) measured by ELISA. **a** Shows an increase of IgGs to both antigens along age groups within each cross-sectional (p trend < 0.001in all four cases). In (**a**) and (**b**) levels between years were compared by Wilcoxon rank-sum test (adjusted p-values for multiple testing by the Holm approach < 0.05 are shown)

Levels of IgG against AMA-1 and MSP-1_42_ were higher in 2013 compared to 2010 (p < 0.001 for both IgGs) mirroring the MTI trends between 2010 and 2013, and increased with age within each cross-sectional (p trend < 0.001 in all four cases), reflecting the continuous exposure to *P. falciparum* infection (Fig. 1a). When stratifying by *P. falciparum* infection during the survey, similar trends were observed in the infected and uninfected populations with overall higher IgG levels in the infected subjects (Fig. 1b).

### Description of the subset of participants included in this study

Among infected participants, there were no differences in age median or distribution of age groups between years, while in 2013 the non-infected individuals were younger than in 2010, with more subjects below 10 years old and less above 20 years old (Table 2). Participants’ distribution between neighbourhoods was also different between years in both groups (Table 2). Among the infected subjects, there were no differences in parasitaemia by qPCR between years (Table 2). In total, there were 11 cases of clinical malaria, 8 (12.3%) in 2013 and 3 (3.1%) in 2010 (p = 0.017).

**Table 2:** Description of *Plasmodium falciparum* infected and uninfected study participants (defined by qPCR)

	Infected (by qPCR)			Uninfected (by qPCR)		
	2010	2013	p-value	2010	2013	p-value
N	96	65		138	78	
Age [median (IQR)]	9.79 (3.61–35.89)	10.06 (3.31–27.64)	0.462	28.4 (5.86–64.03)	7.18 (3.28–36.38)	0.002
Age group (%)			0.737			0.016
< 2 years	8 (8.3)	10 (15.4)		15 (10.9)	14 (17.9)	
2–5 years	29 (30.2)	16 (24.6)		15 (10.9)	15 (19.2)	
5–10 years	12 (12.5)	6 (9.2)		15 (10.9)	14 (17.9)	
10–20 years	15 (15.6)	12 (18.5)		0 (0.0)	1 (1.3)	
20–60 years	23 (24.0)	16 (24.6)		45 (32.6)	18 (23.1)	
> 60 years	9 (9.4)	5 (7.7)		48 (34.8)	16 (20.5)	
Male (%)	42 (43.8)	31 (47.7)	0.632	49 (35.5)	33 (42.3)	0.381
Anaemia (%)	47 (49.0)	28 (43.1)	0.521	79 (57.2)	39 (50.0)	0.322
Area (%)			0.002			< 0.001
Ilha Josina	19 (19.8)	19 (29.2)		23 (16.7)	4 (5.1)	
Malavele	7 (7.3)	9 (13.8)		12 (8.7)	11 (14.1)	
Manhiça	26 (27.1)	11 (16.9)		45 (32.6)	25 (32.1)	
Maragra	30 (31.2)	6 (9.2)		51 (37.0)	15 (19.2)	
Palmeira	11 (11.5)	16 (24.6)		4 (2.9)	17 (21.8)	
Taninga	3 (3.1)	4 (6.2)		3 (2.2)	6 (7.7)	
Par. dens. qPCR (parasites/µL) [median (IQR)]	10.46 (1.61–102.94)	7.61 (1.43–299.84)	0.999	NA		NA
Clinical malaria (%)			0.026	NA		NA
No	93 (96.9)	55 (84.6)				
Yes	3 (3.1)	8 (12.3)				
NA	0 (0.0)	2 (3.1)				

Continuous variables were analysed using the non-parametric Wilcoxon rank-sum test. Comparisons between groups for categorical variables were done using Fisher’s exact test. Parasite densities were log_10_ transformed

### Cytokine, chemokine and growth factor profiles differed between 2010 and 2013

**Fig. 2:**
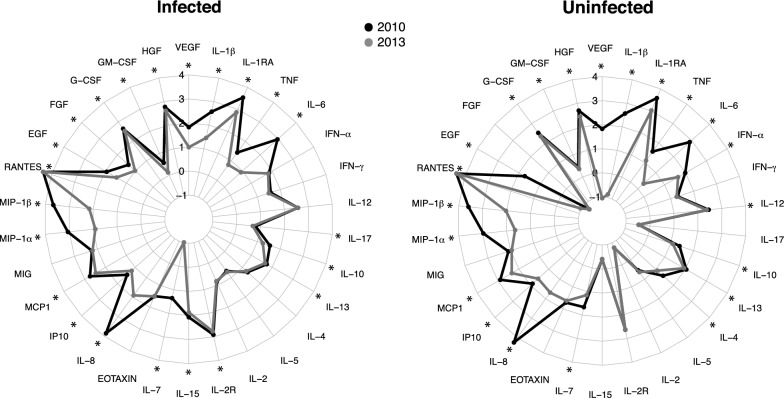
Differences in analyte concentrations between 2010 and 2013 stratified by infection. Radar charts representing the medians of each analyte concentration (log_10_ pg/mL) in 2010 and 2013 and stratifying in infected and uninfected subjects. Levels into each group have been compared between years by Wilcoxon rank-sum test and p-values were adjusted for multiple testing by the Benjamini–Hochberg approach. Statistically significant differences between years are highlighted with an asterisk

The main aim of the study was to evaluate differences in cellular analyte concentrations between years of different MTI in *P. falciparum*-infected and uninfected individuals separately, as differences by MTI were expected to be altered by infection. Overall, 25 analytes out of 30 had higher levels in 2010 compared to 2013 in infected and/or uninfected volunteers, except for RANTES that showed an opposite pattern (Fig. 2, Additional file 2 and Additional file 3). The analytes that increased in 2010 were: the pro-inflammatory cytokines IL-1β, the IL-1 inhibitor IL-1RA, TNF and IL-6; the anti-inflammatory cytokine IL-10; the T_H_2 cytokine IL-13; the regulatory cytokine IL-7; the chemokines IL-8, IP-10, MCP-1, MIP-1α and MIP-1β; and the growth factors EGF, G-CSF, GM-CSF, HGF and VEGF. The regulatory or T_H_1-related cytokine IL-15, the T_H_1 cytokine receptor IL-2R, the anti-inflammatory cytokine IL-17 and the growth factor FGF were higher in 2010 only in the infected individuals (Fig. 2 and Additional file 2). The pro-inflammatory cytokine IFN-α, the T_H_1 cytokine IL-12 and the T_H_2 IL-4 were higher in 2010 only in the non-infected subjects (Fig. 2 and Additional file 3).

**Fig. 3:**
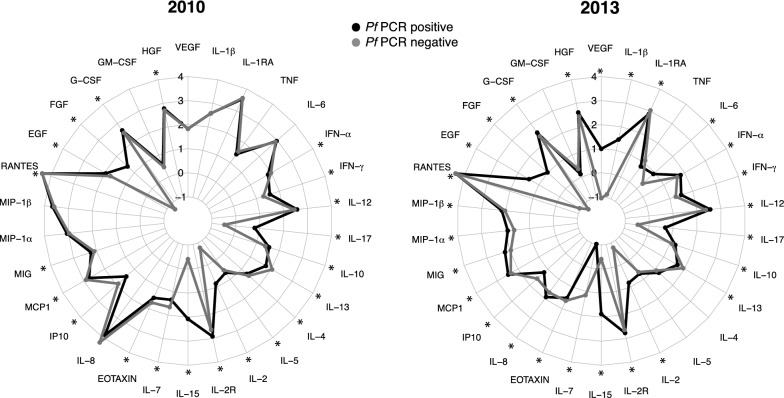
Differences in analytes concentrations between *P. falciparum*-infected and uninfected subjects stratified by year. Radar charts representing the medians of each analyte concentration (log_10_ pg/mL) in infected and uninfected subjects and stratifying by year. Levels between infected and uninfected subjects into each year have been compared by Wilcoxon rank-sum test and p-values were adjusted for multiple testing by the Benjamini–Hochberg approach. Statistically significant differences between infected and uninfected subjects are highlighted with an asterisk

Levels between infected and non-infected individuals were also compared. As expected, *P. falciparum* infection was associated with higher levels of several cytokines, chemokines and growth factors in both years (Fig. 3): IFN-γ, IL-12, IL-17, IL-10, IL-2, IL-2R, IL-15, MIG, EGF, FGF, G-CSF and HGF. However, some markers were only affected in 2013: IL-1β, IL-6, IL-8, MIP-1α, MIP-1β and VEGF; and IL-5 only in 2010. There were also some analytes that were lower in the infected vs the uninfected individuals: IL-13, IL-7, eotaxin, IP-10 and RANTES in both years; IL-4 in 2010; and IL-1RA in 2013. IFN-α and MCP1 showed opposite patterns depending on the year. Additional file 1: Table S2 shows the effect of the infection on analyte levels and the interaction with year.

### Correlations of cytokines, chemokines and growth factors with antibodies differ between 2010 and 2013

The correlations of cellular analyte concentrations with malaria antibodies as markers of exposure were explored. When considering data from 2010 and 2013 together (Table 3), the overall trend showed an inverse correlation of several cytokines and chemokines with antibodies to AMA-1 and MSP-1 in the univariable and multivariable models. In the univariable models, IL-13, IL-1RA, IP-10 and IL-2R negatively correlated with both antibodies, IL-12 was negatively correlated with IgG to AMA-1, and VEGF negatively correlated with IgG to MSP-1. The correlations of AMA-1 and MSP-1 IgG levels with IFN-α, AMA-1 IgG with VEGF and MSP-1 IgG with MCP-1 were different in 2010 compared to 2013 (Table 3, Additional file 4).

**Table 3:** Univariable and multivariable linear regression models to assess the effect of IgGs to AMA-1 and MSP-1 on the cellular immune mediator concentrations and interaction with year

Analyte	IgG to AMA-1					IgG to MSP-1				
	Univariable		Multivariable & interaction with year			Univariable		Multivariable & interaction with year		
	Coefficient (CI)	p-value	Coefficient (CI)	p-value	p-value Int	Coefficient (CI)	p-value	Coefficient (CI)	p-value	p-value Int
FGF	− 0.024 (− 0.048, 0)	*0.047*	− 0.068 (− 0.261, 0.125)	0.486	0.636	− 0.019 (− 0.036, − 0.002)	*0.027*	− 0.016 (− 0.162, 0.13)	0.829	0.996
IL-1β	− 0.026 (− 0.054, 0.002)	0.07	− 0.046 (− 0.235, 0.144)	0.636	0.658	− 0.02 (− 0.037, − 0.003)	*0.022*	0.004 (− 0.124, 0.132)	0.956	0.954
G-CSF	− 0.002 (− 0.009, 0.004)	0.419	− 0.046 (− 0.091, 0)	*0.05*	0.058	− 0.002 (− 0.006, 0.002)	0.359	− 0.026 (− 0.062, 0.009)	0.146	0.155
IL-10	− 0.009 (− 0.035, 0.017)	0.511	− 0.098 (− 0.29, 0.094)	0.315	0.331	0.001 (− 0.017, 0.02)	0.886	− 0.122 (− 0.272, 0.029)	0.112	0.086
IL-13	− 0.017 (− 0.02, − 0.007)	0.001*	− 0.029 (− 0.108, 0.05)	0.473	0.75	− 0.012 (− 0.019, − 0.004)	0.002*	0.002 (− 0.06, 0.063)	0.954	0.707
IL-6	− 0.024 (− 0.071, 0.024)	0.335	0.014 (− 0.235, 0.262)	0.914	0.984	− 0.033 (− 0.067, 0.001)	0.055	− 0.015 (− 0.22, 0.19)	0.888	0.837
IL-12	− 0.007 (− 0.012, − 0.002)	0.004*	− 0.045 (− 0.083, − 0.008)	*0.018*	*0.042*	− 0.003 (− 0.007, 0)	0.06	− 0.01 (− 0.041, 0.021)	0.531	0.639
RANTES	0.005 (0.001, 0.01)	*0.014*	0.015 (− 0.016, 0.047)	0.338	0.501	0.002 (− 0.001, 0.005)	0.124	− 0.004 (− 0.029, 0.021)	0.769	0.689
Eotaxin	− 0.011 (− 0.026, 0.004)	0.141	− 0.103 (− 0.216, 0.01)	0.075	0.106	− 0.005 (− 0.015, 0.006)	0.385	− 0.037 (− 0.126, 0.052)	0.414	0.458
IL-17	− 0.019 (− 0.058, 0.02)	0.331	0.043 (− 0.258, 0.344)	0.778	0.687	0.004 (− 0.024, 0.033)	0.778	− 0.135 (− 0.37, 0.099)	0.256	0.229
MIP-1α	0.005 (− 0.014, 0.025)	0.591	− 0.027 (− 0.13, 0.076)	0.611	0.427	− 0.008 (− 0.021, 0.006)	0.269	− 0.058 (− 0.141, 0.024)	0.166	0.122
GM-CSF	− 0.021 (− 0.083, 0.041)	0.5	− 0.616 (− 1.092, − 0.141)	*0.011*	*0.013*	− 0.017 (− 0.062, 0.028)	0.451	− 0.336 (− 0.747, 0.075)	0.109	0.113
MIP-1β	− 0.003 (− 0.021, 0.015)	0.76	− 0.03 (− 0.111, 0.051)	0.463	0.365	− 0.013 (− 0.026, − 0.001)	*0.039*	− 0.027 (− 0.092, 0.037)	0.409	0.385
MCP-1	− 0.00 (− 0.01, 0.005)	0.504	− 0.061 (− 0.11, − 0.012)	*0.015*	*0.014*	− 0.007 (− 0.012, − 0.001)	*0.017*	− 0.062 (− 0.1, − 0.024)	0.001*	0.002*
IL-15	− 0.004 (− 0.018, 0.011)	0.626	0.064 (− 0.051, 0.18)	0.272	0.245	0.003 (− 0.007, 0.013)	0.531	0.033 (− 0.058, 0.125)	0.475	0.518
EGF	− 0.01 (− 0.027, 0.008)	0.27	− 0.009 (− 0.141, 0.123)	0.889	0.959	− 0.006 (− 0.017, 0.005)	0.289	0.008 (− 0.087, 0.103)	0.871	0.852
IL-5	− 0.036 (− 0.081, 0.009)	0.121	− 0.11 (− 0.46, 0.241)	0.539	0.685	− 0.008 (− 0.041, 0.024)	0.613	− 0.285 (− 0.557, − 0.013)	*0.04*	*0.048*
HGF	− 0.005 (− 0.013, 0.002)	0.16	− 0.045 (− 0.099, 0.009)	0.101	0.129	− 0.001 (− 0.006, 0.004)	0.632	− 0.025 (− 0.067, 0.017)	0.237	0.212
VEGF	− 0.027 (− 0.049, − 0.005)	*0.018*	*0.242 (0.111, 0.373)*	<0.001*	<0.001*	− 0.022 (− 0.037, − 0.006)	0.006*	0.096 (− 0.011, 0.203)	0.078	0.055
IFN-γ	0.002 (− 0.009, 0.013)	0.747	− 0.014 (− 0.098, 0.069)	0.735	0.7	0.004 (− 0.004, 0.012)	0.31	− 0.033 (− 0.1, 0.034)	0.33	0.272
IFN-α	− 0.005 (− 0.012, 0.002)	0.194	− 0.09 (− 0.139, − 0.04)	<0.001*	0.001*	− 0.005 (− 0.009, 0)	0.067	− 0.068 (− 0.106, − 0.03)	0.001*	0.001*
IL-1RA	− 0.01 (− 0.018, − 0.003)	0.007*	0.025 (− 0.018, 0.068)	0.261	0.145	− 0.008 (− 0.013, − 0.002)	0.004*	0.034 (− 0.001, 0.07)	0.055	*0.036*
TNF	0.001 (− 0.029, 0.03)	0.953	− 0.056 (− 0.264, 0.151)	0.592	0.527	− 0.013 (− 0.034, 0.009)	0.242	− 0.089 (− 0.255, 0.077)	0.292	0.287
IL-2	0.006 (− 0.03, 0.042)	0.736	− 0.006 (− 0.29, 0.279)	0.968	0.936	0.018 (− 0.008, 0.043)	0.178	0.005 (− 0.221, 0.23)	0.969	0.907
IL-7	− 0.009 (− 0.024, 0.006)	0.249	− 0.027 (− 0.155, 0.102)	0.68	0.781	− 0.002 (− 0.014, 0.009)	0.678	0.082 (− 0.023, 0.186)	0.126	0.114
IP-10	− 0.037 (− 0.054, − 0.021)	<0.001*	− 0.087 (− 0.205, 0.032)	0.152	0.377	− 0.02 (− 0.031, − 0.008)	0.001*	− 0.07 (− 0.163, 0.024)	0.146	0.248
IL-2R	− 0.016 (− 0.024, − 0.009)	<0.001*	− 0.019 − 0.079, 0.04)	0.52	0.909	− 0.008 (− 0.013, − 0.002)	0.005*	0.008 (− 0.04, 0.055)	0.751	0.53
MIG	− 0.01 (− 0.021, 0.001)	0.079	− 0.086 (− 0.171, − 0.002)	*0.046*	0.072	− 0.003 (− 0.011, 0.005)	0.49	− 0.038 (− 0.106, 0.03)	0.275	0.293
IL-4	− 0.014 (− 0.031, 0.002)	0.085	− 0.118 (− 0.239, 0.003)	0.056	0.081	− 0.007 (− 0.019, 0.004)	0.201	− 0.08 (− 0.173, 0.013)	0.092	0.104
IL-8	0.006 (− 0.02, 0.031)	0.657	0.052 (− 0.073, 0.177)	0.413	0.595	− 0.016 (− 0.034, 0.003)	0.091	− 0.019 (− 0.118, 0.081)	0.712	0.644

p-values < 0.05 are in italic

* p-values < 0.05 after adjusted for multiple comparisons by the Benjamini–Hochberg approach

### Year-dependent effects of age, sex and neighbourhood on analyte concentrations

#### Effect of age

**Fig. 4:**
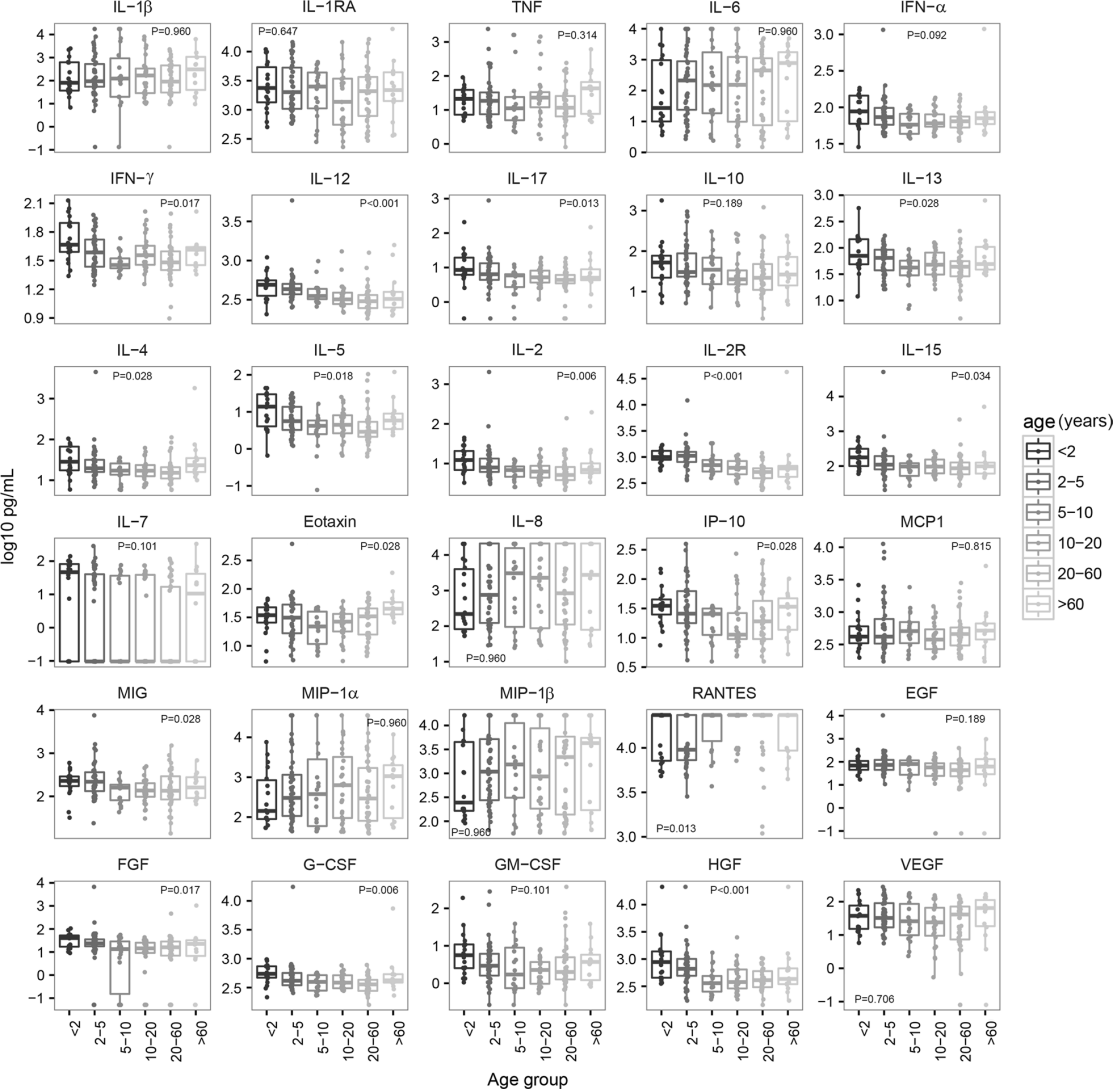
Differences in concentrations of cellular immune mediators between age groups in *P. falciparum*-infected subjects from both years combined. Box plots representing the median and interquartile range of each analyte concentration (log_10_ pg/mL) in infected subjects stratified by age group. Levels between age groups have been compared by Kruskal–Wallis test. *P*-values were adjusted for multiple testing using the Benjamini–Hochberg approach

**Fig. 5:**
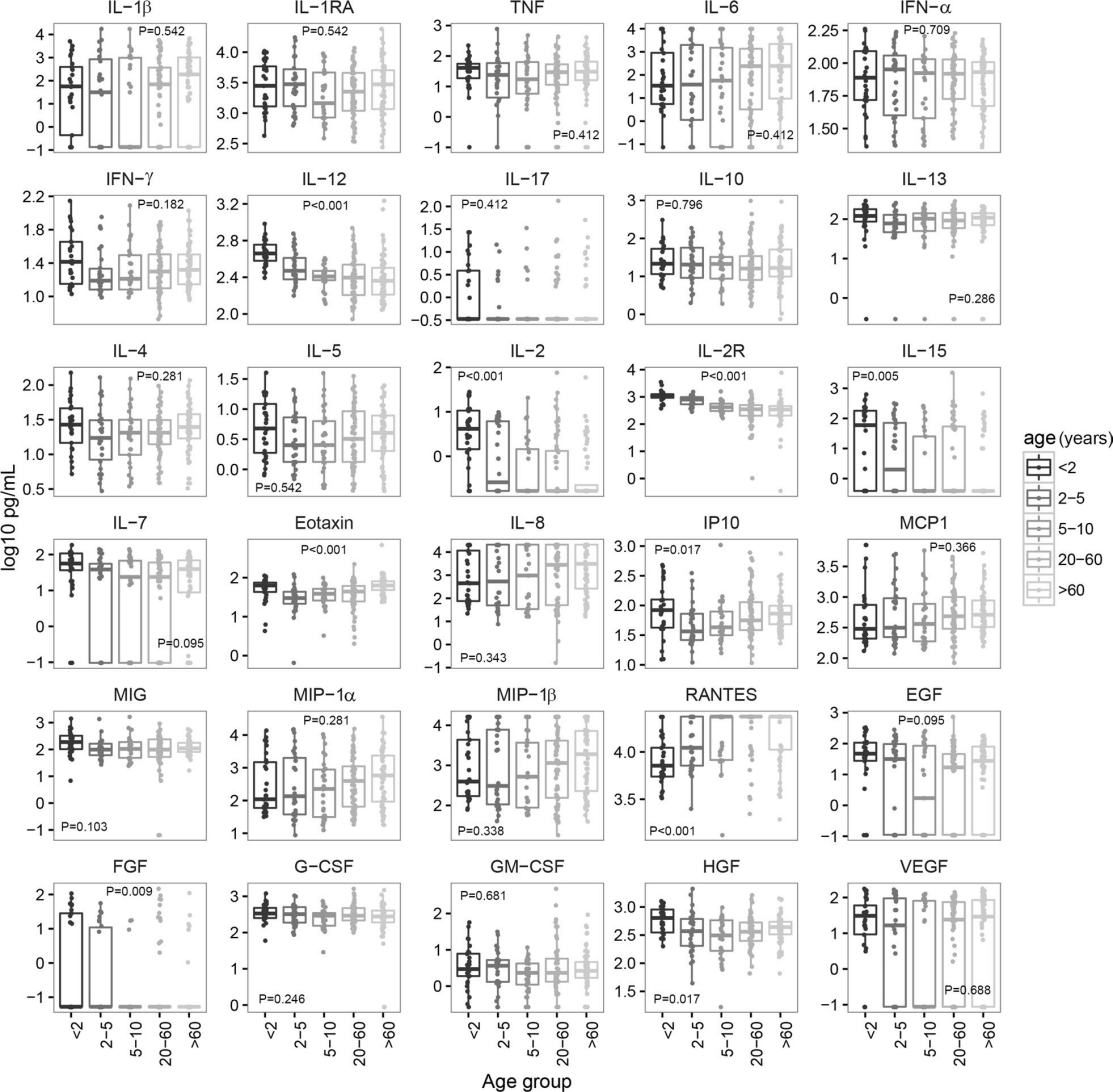
Differences in concentrations of cellular immune mediators between age groups in uninfected subjects from both years combined. Box plots representing the median and interquartile range of each analyte concentration (log_10_ pg/mL) in uninfected subjects stratified by age group. Levels between age groups have been compared by Kruskal–Wallis test. *P*-values were adjusted for multiple testing using the Benjamini–Hochberg approach

The effect of age on the cytokine, chemokine and growth factor responses was assessed, with particular interest in the children and the elderly who have a developing and a senescent immune system, respectively. Age had a statistically significant effect on some analyte concentrations in infected and uninfected individuals, with different patterns depending on the analyte and infection status (Figs. 4 and 5). Levels of IL-12, IL-2, IL-2R, IL-15, FGF and HGF decreased with age in both groups. IFN-γ, IL-17, IL-13, IL-4, IL-5, MIG and G-CSF only decreased in the infected subjects, with some of the analytes slightly increasing in the elderly. Although not statistically significant, other analytes also showed a decreasing trend with respect to age in the infected group like IFN-α, IL-10, IL-15 and GM-CSF. The most marked drop in concentrations was always between age 2 and 10 years old. RANTES levels increased with age in both groups. Eotaxin and IP-10 profiles showed a U-shape with higher values in the groups < 2 years and > 60 years old. A trend of U-shape was also observed for some other analytes like the T_H_2 cytokines IL-4, IL-5, IL-13 and the G-CSF in the infected group. Overall, the children and the elderly showed opposite patterns for some cytokines but similar for others, reflecting the intrinsic characteristics of the immune system in these two age groups and suggesting a process of immunosenescence in the older one.

The interaction of age with year on analyte concentrations was assessed, and statistically significant interactions were found for eotaxin and VEGF in infected individuals, and IL-6, MCP-1, EGF, G-CSF, HGF and VEGF in uninfected individuals (Additional file 1: Table S3 and Additional file 5), however, after correcting by multiple testing significance disappeared (Additional file 1: Table S3).

#### Effect of sex

Sex did not have an effect on analyte concentrations in either infected or uninfected subjects (Additional file 1: Table S4). However, an interaction of sex with year was found for RANTES and GM-CSF concentrations in infected individuals (p-values = 0.002 and 0.041, respectively), although the significance was only maintained for RANTES after adjusting for multiple comparisons. RANTES presented higher levels in males compared to females in 2010, but the opposite was observed in 2013 (Additional file 6). Sex and year interactions were found in uninfected individuals for TNF, IFN-γ and IL-4, although they were not significant after adjusting for multiple testing (Additional file 1: Table S4 and Additional file 6).

#### Effect of neighbourhood

**Fig. 6:**
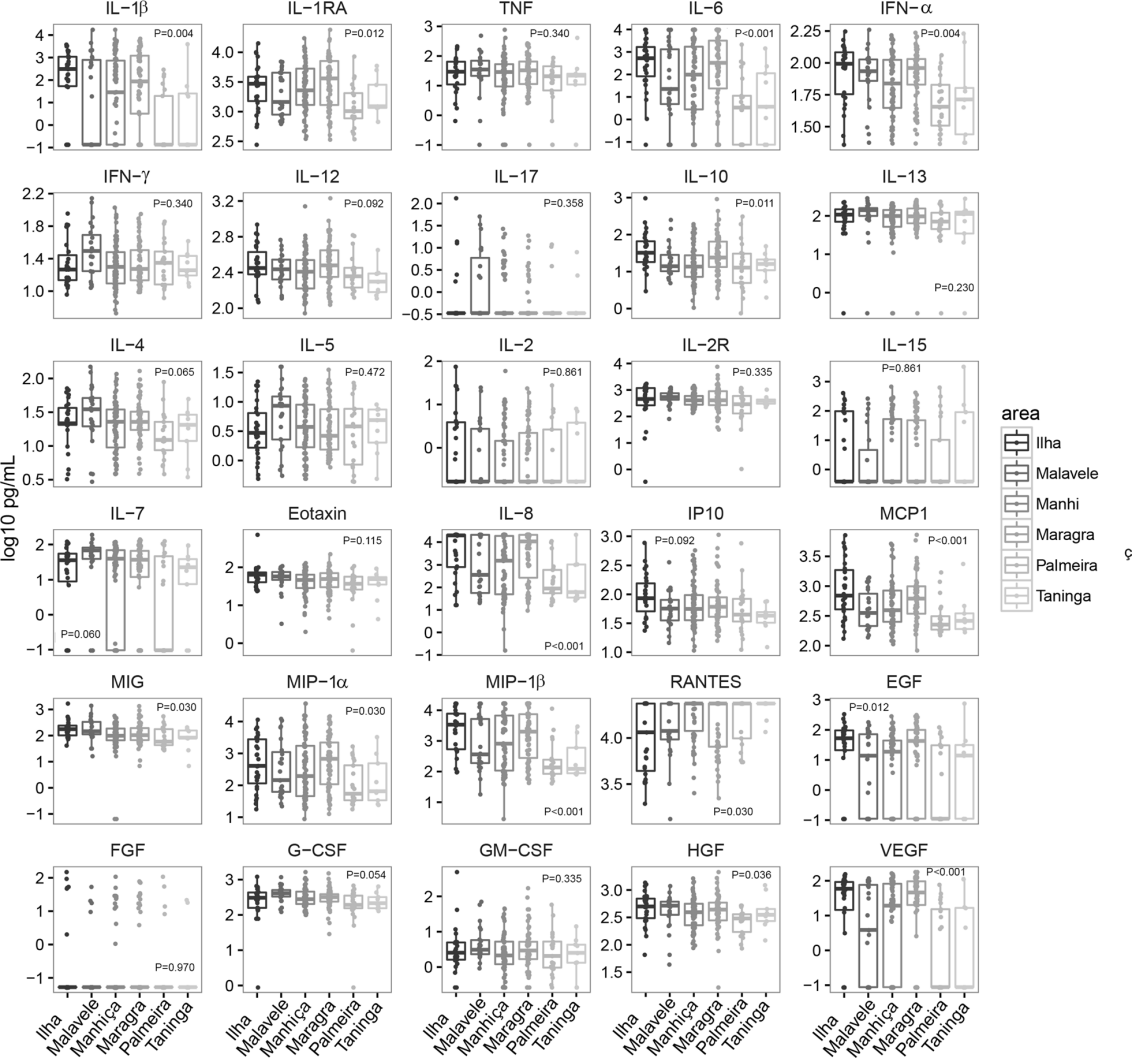
Differences in concentrations of cellular immune mediators between areas in uninfected subjects. Box plots representing the median and interquartile range of each analyte concentration (log_10_ pg/mL) in uninfected subjects stratified by neighbourhood. Levels between areas have been compared by Kruskal–Wallis test. p-values were adjusted for multiple testing using the Benjamini–Hochberg approach

The neighbourhood had an effect on analyte concentrations only in the uninfected subjects (Fig. 6), with lower levels of several of them (IL-1β, IL-1RA, IL-6, IFN-α, IL-8, MCP-1, MIP-1α, MIP-1β and VEGF) in Palmeira and Taninga, and a trend of higher levels in Ilha Josina and Maragra; and the opposite trend for RANTES. No effect was observed in infected individuals (Additional file 7). However, the neighbourhood only interacted with year for IL-5 and FGF in the infected individuals, and the interaction only remained significant for FGF after adjusting by multiple testing (Additional file 1: Table S5).

### Effect of parasitaemia on analyte concentrations

Among *P. falciparum*-infected subjects, parasite density had an effect on different analytes (Table 4). IL-10, IL-2R, IL-12 and MCP-1 increased with higher parasitaemia, and IFN-γ, IL-13, IL-5, IL-7 and eotaxin decreased with increasing parasitaemia (Table 4). The effect only remained significant for IL-10, IL-13 and IL-2R when adjusting for multiple testing. The effect of parasitaemia on IL-10 and GM-CSF was different by year (Table 4 and Additional file 8). However, after adjusting for multiple testing, interaction only remained statistically significant for IL-10, showing a stronger correlation with parasitaemia in 2013 compared to 2010.

**Table 4:** Effect of parasite density (qPCR) on cellular immune mediators concentrations and interaction with year

Analyte	Parasite density by qPCR				
	Coefficient (CI)	p-value	p-value BH	p-value interaction	p-value interaction BH
IL-1β	− 0.076 (− 0.186, 0.034)	0.176	0.377	0.296	0.902
IL-1RA	0.002 (− 0.053, 0.057)	0.945	0.945	0.143	0.860
TNF	− 0.072 (− 0.149, 0.006)	0.069	0.207	0.764	0.916
IL-6	0.017 (− 0.122, 0.157)	0.805	0.894	0.274	0.902
IFN-α	− 0.015 (− 0.041, 0.011)	0.252	0.472	0.883	0.916
IFN-γ	− 0.025 (− 0.049, − 0.001)	*0.045*	0.151	0.470	0.916
IL-12	0.03 (0.007, 0.053)	*0.010*	0.076	0.894	0.916
IL-17	− 0.046 (− 0.1, 0.008)	0.097	0.266	0.409	0.916
IL-10	0.157 (0.098, 0.216)	< *0.001*	<*0.001*	<*0.001*	*0.002*
IL-13	− 0.057 (− 0.095, − 0.018)	*0.004*	*0.040*	0.772	0.916
IL-4	− 0.029 (− 0.074, 0.016)	0.207	0.414	0.912	0.916
IL-5	− 0.07 (− 0.126, − 0.014)	*0.015*	0.090	0.515	0.916
IL-2	− 0.022 (− 0.066, 0.023)	0.338	0.590	0.753	0.916
IL-2R	0.053 (0.021, 0.085)	*0.001*	*0.018*	0.898	0.916
IL-15	− 0.016 (− 0.063, 0.031)	0.506	0.732	0.819	0.916
IL-7	− 0.193 (− 0.354, − 0.032)	*0.019*	0.094	0.785	0.916
Eotaxin	− 0.04 (− 0.079, − 0.002)	*0.040*	0.148	0.179	0.896
IL-8	− 0.053 (− 0.18, 0.075)	0.414	0.653	0.301	0.902
IP-10	0.024 (− 0.027, 0.074)	0.354	0.590	0.799	0.916
MCP1	0.041 (0.002, 0.081)	*0.039*	0.148	0.443	0.916
MIG	0.035 (− 0.011, 0.081)	0.134	0.334	0.276	0.902
MIP-1α	− 0.027 (− 0.128, 0.074)	0.594	0.742	0.500	0.916
MIP-1β	− 0.004 (− 0.107, 0.098)	0.933	0.945	0.093	0.697
RANTES	− 0.01 (− 0.044, 0.024)	0.561	0.732	0.896	0.916
EGF	− 0.015 (− 0.089, 0.059)	0.683	0.820	0.916	0.916
FGF	− 0.007 (− 0.125, 0.112)	0.911	0.945	0.734	0.916
G-CSF	− 0.009 (− 0.035, 0.018)	0.522	0.732	0.366	0.916
GM-CSF	− 0.05 (− 0.117, 0.018)	0.147	0.339	*0.028*	0.424
HGF	0.007 (− 0.035, 0.049)	0.733	0.846	0.712	0.916
VEGF	0.023 (− 0.055, 0.101)	0.560	0.732	0.076	0.697

The effect of parasite density on analytes concentrations was assessed through multivariable linear regressions for each analyte, with analyte concentration as outcome (pg/mL) and parasitaemia as the predictor variable. Interaction tests were performed to determine if there was an interaction of parasite density with year on the analyte levels. BH: p-values adjusted for multiple testing by Benjamini–Hochberg

*P*-values < 0.05 are in italic

*CI* confidence intervals

### Adjusted effect of year on cytokine, chemokine and growth factor concentrations

The effect of the year on analyte concentrations was also assessed in regression models adjusting by age and neighbourhood. Results were similar to the unadjusted analyses, with lower levels of almost the same analytes in 2013 compared to 2010 in infected and uninfected volunteers, and RANTES showing an opposite pattern (Table 5). Analytes that were lower in 2013 in infected and non-infected individuals were: the pro-inflammatory cytokines IL-1β, IL-1RA, TNF, IL-6; the T_H_1 cytokine IL-2R; the anti-inflammatory cytokine IL-10; the T_H_2 cytokine IL-13; the regulatory cytokine IL-7; the chemokines IL-8, IP-10, MCP1, MIP-1α and MIP-1β; and the growth factors EGF, G-CSF, GM-CSF, HGF and VEGF. The regulatory or T_H_1-related cytokine IL-15 and the growth factor FGF were lower in 2013 only in the infected subjects. In the non-infected individuals, there were some additional analytes also with lower levels in 2013: the pro-inflammatory cytokine IFN-α, the T_H_1 cytokine IL-12, and the T_H_2 cytokine IL-4.

**Table 5:** Effect of 2013 compared to 2010 on analyte concentrations in infected and uninfected individuals

Analytes	Coefficient (CI)	p-value	p-value BH	Coefficient (CI) adjusted	p-value adjusted*	p-value BH adjusted*
Infected						
IL-1β	− 1.248 (− 1.463, − 1.034)	< *0.001*	< *0.001*	− 1.311 (− 1.543, − 1.079)	< *0.001*	< *0.001*
IL-1RA	− 0.638 (− 0.743, − 0.534)	< *0.001*	*< 0.001*	− 0.67 (− 0.781, − 0.559)	*< 0.001*	*< 0.001*
TNF	− 0.666 (− 0.842, − 0.49)	*< 0.001*	*< 0.001*	− 0.678 (− 0.867, − 0.489)	*< 0.001*	*< 0.001*
IL-6	− 1.945 (− 2.144, − 1.746)	*< 0.001*	*< 0.001*	− 2.023 (− 2.239, − 1.807)	*< 0.001*	*< 0.001*
IFN-α	− 0.025 (− 0.093, 0.043)	0.470	0.486	− 0.026 (− 0.097, 0.046)	0.481	0.505
IFN-γ	− 0.036 (− 0.099, 0.028)	0.268	0.321	− 0.039 (− 0.103, 0.026)	0.235	0.293
IL-12	− 0.035 (− 0.096, 0.027)	0.265	0.321	− 0.029 (− 0.092, 0.033)	0.358	0.410
IL-17	− 0.147 (− 0.288, − 0.006)	*0.041*	0.057	− 0.149 (− 0.295, − 0.002)	*0.047*	0.064
IL-10	− 0.227 (− 0.391, − 0.063)	*0.007*	*0.010*	− 0.235 (− 0.407, − 0.063)	*0.008*	*0.012*
IL-13	− 0.119 (− 0.22, − 0.017)	*0.022*	*0.031*	− 0.149 (− 0.252, − 0.046)	*0.005*	*0.009*
IL-4	− 0.076 (− 0.193, 0.042)	0.205	0.267	− 0.073 (− 0.196, 0.051)	0.245	0.295
IL-5	− 0.018 (− 0.167, 0.132)	0.815	0.815	− 0.033 (− 0.187, 0.121)	0.673	0.673
IL-2	− 0.064 (− 0.181, 0.052)	0.278	0.321	− 0.056 (− 0.177, 0.066)	0.369	0.410
IL-2R	− 0.126 (− 0.21, − 0.042)	*0.003*	*0.006*	− 0.123 (− 0.202, − 0.043)	*0.003*	*0.005*
IL-15	− 0.182 (− 0.302, − 0.063)	*0.003*	*0.006*	− 0.168 (− 0.294, − 0.043)	*0.009*	*0.013*
IL-7	− 0.819 (− 1.228, − 0.411)	*< 0.001*	*< 0.001*	− 0.858 (− 1.279, − 0.436)	*< 0.001*	*< 0.001*
Eotaxin	− 0.043 (− 0.145, 0.058)	0.401	0.430	− 0.066 (− 0.173, 0.04)	0.221	0.288
IL-8	− 1.769 (− 1.956, − 1.582)	*< 0.001*	*< 0.001*	− 1.868 (− 2.067, − 1.669)	*< 0.001*	*< 0.001*
IP-10	− 0.242 (− 0.369, − 0.116)	*< 0.001*	*0.001*	− 0.227 (− 0.36, − 0.094)	*0.001*	*0.002*
MCP1	− 0.29 (− 0.384, − 0.196)	*< 0.001*	*< 0.001*	− 0.284 (− 0.385, − 0.182)	*< 0.001*	*< 0.001*
MIG	− 0.061 (− 0.181, 0.059)	0.313	0.348	− 0.044 (− 0.169, 0.081)	0.488	0.505
MIP-1α	− 1.14 (− 1.335, − 0.946)	**<** *0.001*	*< 0.001*	− 1.2 (− 1.41, − 0.991)	*< 0.001*	*< 0.001*
MIP-1β	− 1.413 (− 1.565, − 1.26)	*< 0.001*	*< 0.001*	− 1.454 (− 1.622, − 1.287)	*< 0.001*	*< 0.001*
RANTES	0.143 (0.056, 0.23)	*0.001*	*0.003*	0.109 (0.018, 0.201)	*0.019*	*0.027*
EGF	− 0.61 (− 0.778, − 0.442)	*< 0.001*	*< 0.001*	− 0.684 (− 0.858, − 0.511)	*< 0.001*	*< 0.001*
FGF	− 0.754 (− 1.04, − 0.467)	*< 0.001*	*< 0.001*	− 0.935 (− 1.222, − 0.648)	*< 0.001*	*< 0.001*
G-CSF	− 0.1 (− 0.169, − 0.032)	*0.004*	*0.006*	− 0.096 (− 0.167, − 0.026)	*0.008*	*0.012*
GM-CSF	− 0.291 (− 0.462, − 0.12)	*0.001*	*0.002*	− 0.345 (− 0.524, − 0.167)	*< 0.001*	*< 0.001*
HGF	− 0.16 (− 0.268, − 0.053)	*0.004*	*0.006*	− 0.154 (− 0.261, − 0.046)	*0.005*	*0.009*
VEGF	− 0.913 (− 1.057, − 0.769)	*< 0.001*	*< 0.001*	− 0.967 (− 1.118, − 0.816)	*< 0.001*	*< 0.001*
Uninfected						
IL-1β	− 2.586 (− 2.949, − 2.223)	< *0.001*	< *0.001*	− 2.551 (− 2.961, − 2.14)	< *0.001*	< *0.001*
IL-1RA	− 0.501 (− 0.597, − 0.406)	< *0.001*	< *0.001*	− 0.523 (− 0.628, − 0.418)	< *0.001*	< *0.001*
TNF	− 0.684 (− 0.877, − 0.49)	< *0.001*	< *0.001*	− 0.699 (− 0.917, − 0.48)	< *0.001*	< *0.001*
IL-6	− 2.876 (− 3.129, − 2.623)	< *0.001*	< *0.001*	− 2.878 (− 3.162, − 2.595)	< *0.001*	< *0.001*
IFN-α	− 0.291 (− 0.344, − 0.239)	< *0.001*	< *0.001*	− 0.288 (− 0.347, − 0.23)	< *0.001*	< *0.001*
IFN-γ	0.002 (− 0.076, 0.08)	0.960	0.960	− 0.001 (− 0.087, 0.085)	0.975	0.975
IL-12	− 0.129 (− 0.19, − 0.068)	< *0.001*	< *0.001*	− 0.158 (− 0.217, − 0.098)	< *0.001*	< *0.001*
IL-17	0.072 (− 0.085, 0.228)	0.369	0.410	0.054 (− 0.121, 0.229)	0.544	0.628
IL-10	− 0.391 (− 0.53, − 0.251)	< *0.001*	< *0.001*	− 0.376 (− 0.532, − 0.221)	< *0.001*	< *0.001*
IL-13	− 0.264 (− 0.404, − 0.123)	< *0.001*	< *0.001*	− 0.272 (− 0.432, − 0.112)	*0.001*	*0.001*
IL-4	− 0.245 (− 0.342, − 0.148)	< *0.001*	< *0.001*	− 0.233 (− 0.34, − 0.125)	< *0.001*	< *0.001*
IL-5	0.046 (− 0.086, 0.178)	0.493	0.510	0.066 (− 0.083, 0.215)	0.386	0.464
IL-2	0.076 (− 0.13, 0.282)	0.467	0.501	0.012 (− 0.201, 0.224)	0.914	0.946
IL-2R	− 0.12 (− 0.247, 0.007)	0.064	0.087	− 0.195 (− 0.319, − 0.072)	*0.002*	*0.003*
IL-15	− 0.22 (− 0.546, 0.106)	0.184	0.224	− 0.356 (− 0.711, − 0.001)	*0.050*	0.062
IL-7	− 0.868 (− 1.188, − 0.549)	< *0.001*	< *0.001*	− 0.917 (− 1.264, − 0.579)	< *0.001*	< *0.001*
Eotaxin	− 0.081 (− 0.177, 0.015)	0.099	0.129	0.011 (− 0.089, 0.112)	0.824	0.883
IL-8	− 2.096 (− 2.287, − 1.904)	< *0.001*	< *0.001*	− 2.127 (− 2.339, − 1.916)	< *0.001*	< *0.001*
IP-10	− 0.343 (− 0.436, − 0.25)	< *0.001*	< *0.001*	− 0.337 (− 0.441, − 0.233)	< *0.001*	< *0.001*
MCP1	− 0.527 (− 0.615, − 0.44)	< *0.001*	< *0.001*	− 0.515 (− 0.614, − 0.416)	< *0.001*	< *0.001*
MIG	− 0.076 (− 0.215, 0.062)	0.279	0.322	− 0.033 (− 0.186, 0.12)	0.673	0.748
MIP-1α	− 1.267 (− 1.442, − 1.093)	< *0.001*	< *0.001*	− 1.299 (− 1.497, − 1.101)	< *0.001*	< *0.001*
MIP-1β	− 1.484 (− 1.629, − 1.349	< *0.001*	< *0.001*	− 1.478 (− 1.638, − 1.319)	< *0.001*	< *0.001*
RANTES	0.129 (0.05, 0.209)	*0.002*	*0.002*	0.164 (0.087, 0.241)	< *0.001*	< *0.001*
EGF	− 1.555 (− 1.855, − 1.255)	< *0.001*	< *0.001*	− 1.682 (− 2.004, − 1.359)	< *0.001*	< *0.001*
FGF	− 0.201 (− 0.499, 0.098)	0.187	0.224	− 0.336 (− 0.662,− 0.011)	*0.043*	0.056
G-CSF	− 0.211 (− 0.307, − 0.114)	< *0.001*	< *0.001*	− 0.236 (− 0.341, − 0.131)	< *0.001*	< *0.001*
GM-CSF	− 0.195 (− 0.354, − 0.036)	*0.017*	*0.024*	− 0.195 (0.374, − 0.016)	*0.033*	*0.045*
HGF	− 0.29 (− 0.364, − 0.215)	< *0.001*	< *0.001*	− 0.305 (− 0.386, − 0.225)	< *0.001*	< *0.001*
VEGF	− 1.887 (− 2.159, − 1.614)	< *0.001*	< *0.001*	− 1.938 (− 2.235, − 1.641)	< *0.001*	< *0.001*

The effect of year on analyte concentrations was assessed through univariable l and multivariable (adjusted models) separate linear regressions for each analyte, with analyte concentration (pg/mL) as outcome and year (2013 vs 2010) as the predictor variable. The analysis is presented separately for the infected and uninfected subjects. BH: P-values adjusted for multiple testing by Benjamini–Hochberg

*P*-values < 0.05 are in italic

*CI* confidence intervals

* Adjusted by age group and area

## Discussion

In spite of significant reductions in the burden of malaria over the last 10 years [43], scarce monitoring has been done on the impact on malaria immunity. Mozambique is one of the countries with the highest malaria burden in the world, although there are increasing efforts to move towards elimination in southern provinces [44]. Malaria burden decreased in all provinces from a malaria incidence rate over 6 million in 2007 to 3 million in 2011, but an increase was observed again in 2012 reaching near 6 million in 2014 [32, 33, 45]. In this study, the association of shifting epidemiological patterns with the systemic cellular immune profiles, with or without a current infection, was assessed through quantification of several plasma cytokines, chemokines and growth factors.

Previous studies have found that the balance between pro-inflammatory and anti-inflammatory cytokines determines host protection and injury [22, 46, 47], and the same for growth factors and chemokines [48, 49]. Given the importance of these proteins in the immune response and the consequent control of immunopathology, it was hypothesized that they could be importantly affected by changes in MTI. Results from this study show that in both infected and uninfected subjects most of the analytes were at higher concentrations in 2010 than 2013, suggesting the possibility of a blunted cytokine response with higher MTI, which could be associated to host tolerance. Albeit weakly, analytes were negatively associated with antibody markers of malaria exposure, further suggesting a blunted response with higher exposure. For some analytes, the correlations with antibodies differed by year, indicating a different regulation of the cellular response. The higher cytokine, chemokine and growth factor levels in 2010 could be related to a loss of immune tolerance after a decline in MTI, and their lower levels in 2013 could be indicative of a rapid re-establishment of tolerance as a consequence of more continuous exposure. Thus, these cellular immune mediators could be candidate surrogates of MTI and the associated host susceptibility and tolerance deserving further investigation.

Recent studies suggest that repeated exposure to *P. falciparum* leads to the establishment of tolerance [4], which may be associated with the loss and/or altered function of several immune cell types, including γδ T cells [5], αβ T cells [50–52], B cells [53], and myeloid cells [54] that show less proliferation and cytokine production [5]. In addition, malaria exposure also induces changes in the innate immune response [55, 56]. A recent study identifies individuals who are primed to respond favorably to *P. falciparum* infection by controlling inflammatory symptoms (disease tolerance) and parasitaemia [57]. Accordingly, the lower levels of cytokines, chemokines and growth factors observed with increasing MTI could be reflecting a more controlled cellular response attributed to some degree of immuno-tolerance. In agreement with these results, a recent study in children with clinical malaria from Ghana reported a decrease of several cytokines with increasing MTI [58].

Another study showed diminished cytokine response with age in children, in part probably due to increased exposure, which could also be indicative of tolerogenic mechanisms [59]. A decrease in MTI is usually followed by a delayed acquisition of immunity to clinical and severe disease; data from a health facility in one of the higher endemic areas of Manhiça confirm that after years of sustained decrease in malaria incidence, there is an increase in the mean age of clinical malaria events and severe forms of disease [60]. Accordingly, a study on infected pregnant women from Manhiça between 2003 and 2012, showed a decrease of antibodies against *P. falciparum* and an increase of malaria adverse consequences after the decline of MTI [61]. Interestingly, in the present study, the differing cytokine, chemokine and growth factor levels detected in the non-infected individuals between 2010 and 2013 suggests that the impact of MTI could be beyond the response to *P. falciparum* infection at the time of the survey, suggesting that the rate of previous exposures could shape the basal immunological status. This difference in the basal immune system agrees with the observation by Tran et al. [57] of a marked different transcriptomic and cellular profiles between malaria-protected and malaria-susceptible children prior to *Plasmodium* infection. This study also suggests that previous exposure to malaria, and possibly other pathogens or commensals, shape the basal immune system.

In the same line, a previous study by our group in semi-immune African adults, migrants and European travellers, showed that naïve adults had stronger cytokine responses upon infection than semi-immune adults; and that migrants, in the absence of continuous exposure, presented higher concentrations of cytokines and chemokines than semi-immune individuals, but lower than individuals with a first infection [11]. There, increased levels of IL-2, IFN-γ, IL-8 and IL-5 were associated with loss of exposure to *P. falciparum* [11]. Similarly, in the present study, higher levels of IL-8 were observed in 2010. In addition, increased levels of IL-6 and IL-10, and decreased levels of RANTES were detected in 2010; these three cytokines have been associated with severe malaria [22, 47, 62]. Several studies indicate a strong positive role of RANTES against *Plasmodium* species susceptibility [63, 64] and malaria severity [21], being down-regulated in severe malaria compared to uncomplicated malaria [65]. This immunomodulatory role of RANTES reducing the pathogenesis of malaria would agree with this chemokine being at lower levels in the lower MTI period in which a loss of immune-tolerance is hypothesized.

Despite the lower cellular immune mediators observed in 2013, infected individuals showed higher levels of several cytokines and chemokines compared to the uninfected ones, differences that were not observed in 2010; among them, the pro-inflammatory cytokines IFN-α, IL-1β and IL-6, the chemokines IL-8, MCP-1, MIP-1α and MIP-1β and the growth factor VEGF. This may be related to the lower basal levels of cellular immune mediators in 2013 compared to 2010. In 2010, basal levels of cellular mediators were so high that infections might have not been able to induce further increases; whereas in 2013, cytokine, chemokine and growth factor basal levels were low and infections increased them significantly, though still below the basal levels from 2010. This observation supports the hypothesis of a more controlled, non-harmful cellular immune response upon re-infection in 2013.

Despite the overall decrease in cellular immune mediators in 2013, a higher prevalence of clinical malaria (12%) was registered compared to 2010 (3%) among the infected individuals, similar to the prevalence in the surveys (18/162 vs 3/106). However, the total number of clinical malaria cases was low, and clinical malaria depends not only on tolerogenic responses but also on anti-parasite immunity. During lower MTI periods there is a delay in the acquisition of immunity in younger children and a reduction in premunition in older children and adults; thus, in the context of a sudden increase of MTI, anti-parasite immunity may be acquired slower than tolerogenic responses. It is likely that a higher number of clinical cases would have been observed in 2013 had some level of tolerance not been developed.

As expected, among the infected individuals the level of parasitaemia affected several analytes, some of them similar to previous reports [11, 66]. Interestingly, a different effect of parasitaemia on IL-10 and GM-CSF was found depending on the year, reinforcing the idea of different cellular immune responses at different MTIs. A different effect of parasitaemia on cytokine levels depending on MTI was also recently reported [58], with several cytokines correlating with parasitaemia only in the lower MTI area.

Age-related differences in immunity are suggested to also explain the different susceptibility to malaria disease in children vs adults [67] independently of cumulative exposure [8, 68]. Previous studies showed that adult newcomers into a hyperendemic area developed NAI relatively quickly while their children remained susceptible, suggesting that age is relevant for NAI development. NAI against malaria may also be diminished at older ages because of changes in the immune system, known as immunosenescence, which contribute to make elderly more susceptible to infections, cancer and autoimmunity. For example, there is a decline in the output of regulatory T cells after the age of 50 [69, 70] and altered cytokine levels have been observed [71, 72]. How immunosenescence may affect the acquired protection against malaria is still unknown, and the impact of changing MTI could be different. Interestingly, results from this study show that different cellular immune mediators present diverse age patterns, and some were different in different MTI periods while others not. For example, the T_H_1 cytokines IL-2, IL-12 and IL-2R and the T_H_1 related IL-15 decreased continuously with age; while eotaxin, IP-10 and the T_H_2 cytokines IL-13, IL-4 and IL-5 presented a U-shape, i.e. higher levels in children and the elderly and lower levels in young adults. Previous studies have shown that some cytokines increase in the elderly [72], while others seem to decrease [70, 73], but none of the cytokines were the same observed in this study. A U-shape suggests similarities between the children and elderly immune systems. High levels of some analytes in the age 1-2 years old group could be related to a lower immunity due to previous lack of exposure to malaria, but also to the intrinsic different characteristics of the immune system in early life. High levels of some analytes in the elderly could be related to the process of immunosenescence that may cause loss of anti-disease immunity, therefore behaving similar to a first infection or like a less exposed population presenting higher cellular responses upon re-challenge [74].

Previous studies have also shown that the severity of malaria infection differs between males and females [75], with men developing more severe parasitaemia and pathology than women [76]. In addition, it has been described that in general women produce more intense humoral and cell-mediated immune responses than males [77, 78]. However, no effect of sex was observed on the analyte concentrations in either infected or uninfected subjects, although a sex interaction was found with year for TNF, IFN-γ, IL-4, RANTES and GM-CSF, the impact of which is difficult to interpret. MTI is also expected to present spatial heterogeneities due to social and natural factors [79] but here the neighbourhood did not have an effect on the association between year and cytokine, chemokine or growth factor levels.

There are some limitations in this study that could have affected the results. First of all, the different storage times for plasma samples collected in 2010 vs samples collected in 2013, which may have differently affected the cytokine concentrations. However, storage time tends to decrease the concentration of most of the cytokines [80]. Therefore, if there was any general storage effect, it would have been in the opposite direction of what was observed. Secondly, total cytokines in plasma samples were measured instead of cytokines produced by peripheral blood mononuclear cells (PBMCs) upon in vitro stimulations with malaria antigens, which limits the interpretation of malaria-specific responses. However, PBMCs were not available. Third, many comparisons were performed and despite adjusting for multiple testing, some of the significant differences may be false positives. Nevertheless, many consistent and biologically feasible associations of cellular mediators with MTI were found. Fourth, the HIV serostatus of the study participants was not known, and the study area has a high HIV prevalence [81] which may be affecting the results. Finally, it is important to keep in mind that the average rainfall before 2013 survey doubled that before 2010 survey, therefore exposure to infections other than malaria or hydration of study subjects may also vary between years, affecting the systemic homeostatic profile of the inhabitants and their basal immune activation and analyte concentrations, thus it may have influenced or confounded the results. However, the higher *P. falciparum* IgG levels in 2013 compared to 2010 across all age groups and negative correlations with cellular immune mediators suggests that *P. falciparum* exposure could be driving the changes described. Future studies should have a longitudinal design and comprise different MTI periods to address the role of cellular immune mediators as surrogates of MTI and tolerance/susceptibility to malaria. In addition, controlling for other factors influencing the basal immune system would also be important. Understanding the mechanisms of longevity of immunity and tolerance may help in developing approaches to promote long-lasting anti-disease immunity, even after sustained interrupted exposure, which could be used in areas of low MTI and in elimination campaigns.

## Conclusions

Cytokine, chemokine and growth factor profiles varied between years of different MTIs in *P. falciparum* infected and non-infected individuals, which could be related to a loss of immune tolerance when MTI declines and a rapid re-establishment of immune tolerance after an increase in *P. falciparum* exposure. This finding warrants further investigations on plasma cellular immune mediators as potential surrogate candidates of MTI and the associated host susceptibility and tolerance.

## Supplementary information

**Additional file 1: Table S1**. Monthly mean rainfall in the study area in the 5 months previous to the 2010 and 2013 surveys. **Table S2.** Effect of infection on analyte concentrations and the interactions with year. **Table S3.** Interaction of age with year on analyte levels in infected and uninfected individuals. **Table S4.** Effect of sex on analyte concentrations and the interactions with year in infected and uninfected individuals. **Table S5.** Interaction of neighborhood with year on analyte levels in infected and uninfected individuals.

**Additional file 2.** Differences in cellular immune mediator concentrations between 2010 (lower MTI) and 2013 (higher MTI) in *P. falciparum*-infected subjects. Box plots representing the median and interquartile range of each analyte concentration (log_10_ pg/mL) in infected subjects recruited in 2010 and in 2013. Levels between both years were compared by Wilcoxon rank-sum test and p-values were adjusted for multiple testing by Benjamini–Hochberg approach.

**Additional file 3.** Differences in cellular immune mediator concentrations between 2010 (lower MTI) and 2013 (higher MTI) in uninfected subjects. Box plots representing the median and interquartile range of each analyte concentration (log_10_ pg/mL) in uninfected subjects recruited in 2010 and in 2013. Levels between both years were compared by Wilcoxon rank-sum test and p-values were adjusted for multiple testing by the Benjamini–Hochberg approach.

**Additional file 4.** Correlations of VEGF, MCP-1 and IFN-α with *P. falciparum* antibody levels stratified by year. Data are presented in scatter plots with trend lines, with analytes concentrations in the Y axes and antibody levels in the X axes. R^2^ and p-values were obtained through linear regression models. Only analytes that had a statistically significant interaction with year for the correlation with antibodies are shown.

**Additional file 5.** Cellular immune mediator concentrations in 2010 (low MTI) and 2013 (high MTI) stratified by age group. Box plots representing the median and interquartile range of analytes concentrations (log_10_ pg/mL) in *P. falciparum* infected (a) and uninfected (b) subjects. Only analytes in which age and year had a significant *p*-value for the interaction test (before correcting for multiple testing) are shown.

**Additional file 6.** Cellular immune mediator concentrations in 2010 (low MTI) and 2013 (high MTI) stratified by sex. Box plots representing the median and interquartile range of analyte concentrations (log_10_ pg/mL) in *P. falciparum* infected (a) and uninfected (b) subjects. Only analytes in which sex and year had a significant p-value for the interaction test (before correcting for multiple testing) are shown.

**Additional file 7.** Differences in cellular immune mediator concentrations between areas in *P. falciparum* infected subjects. Box plots representing the median and interquartile range of each analyte concentration (log_10_ pg/mL) in infected subjects stratified by neighborhood. Levels between areas have been compared by Kruskal–Wallis test.

**Additional file 8.** Effect of parasitema on IL-10 and GM-CSF concentrations stratified by year. Scatter plots with trend line representing the distribution of analytes concentration by parasitemia stratified by year. Only analytes in which parasitemia and year had a significant p-value for the interaction test (before correcting for multiple testing) are shown.

## Data Availability

The datasets used and/or analysed during the current study are available from the corresponding author on reasonable request.

## Abbreviations

MTI: malaria transmission intensity
NAI: naturally acquired immunity
IFN: interferon
IL: interleukin
TNF: tumour necrosis factor
RANTES: regulated upon activation, normal T cell expressed, and secreted
qPCR: real-time quantitative PCR
ELISA: enzyme-linked immunosorbent assay
MSP: merozoite surface protein
AMA: apical membrane antigen
RT: room temperature
PBS: phosphate buffered saline
T-PBS: Tween-phosphate buffered saline
HRP: horseradish peroxidase
OPD: ortho-phenylenediamine
OD: optical density
EGF: epidermal growth factor
FGF: fibroblast growth factor
G-CSF: granulocyte colony-stimulating factor
GM-CSF: granulocyte-macrophage colony-stimulating factor
HGF: hepatocyte growth factor
VEGF: vascular endothelial growth factor
IP-10: IFN-γ induced protein
MCP-1: monocyte chemoattractant protein
MIG: monokine induced by IFN-γ
MIP: macrophage inflammatory protein
T_H_: T helper lymphocyte
PBMC: peripheral blood mononuclear cells
