# Supplementary material for: Changing plasma cytokine, chemokine and growth factor profiles upon differing malaria transmission intensities
Source: Malar J. 2019 Dec 5;18:406. doi: 10.1186/s12936-019-3038-x (PMC6896751; doi:10.1186/s12936-019-3038-x)
Supplement: Supplementary file 1 — Additional file 1: Table S1. Monthly mean rainfall in the study area in the 5 months previous to the 2010 and 2013 surveys. Table S2. Effect of infection on analyte concentrations and the interactions with year. Table S3. Interaction of age with year on analyte levels in infected and uninfected individuals. Table S4. Effect of sex on analyte concentrations and the interactions with year in infected and uninfected individuals. Table S5. Interaction of neighborhood with year on analyte levels in infected and uninfected individuals. [file 12936_2019_3038_MOESM1_ESM.docx]

## Additional Tables

## Table S1. Monthly mean rainfall in the study area in the 5 months previous to the 2010 and 2013 surveys. Months of recruitment in each survey are in bold. Data collected from CHIRPS data set, <http://chg.geog.ucsb.edu/data/chirps/>.

| **Year** | **Month** | **Mean rain (mm)** |
| --- | --- | --- |
| 2009 | September | 21.69 |
|  | October | 29.78 |
|  | November | 100.43 |
|  | December | 44.03 |
| 2010 | January | 114.83 |
|  | **February** | 46.25 |
|  | **March** | 62.06 |
| 2012 | September | 104.41 |
|  | October | 144.99 |
|  | November | 58.55 |
|  | December | 92.87 |
| 2013 | January | 224.44 |
|  | **February** | 59.12 |
|  | **March** | 53.36 |

**Table S2. Effect of infection on analyte concentrations and the interactions with year.** The effect of infection (detected by qPCR) on analytes levels was assessed through univariate or multivariable (adjusted models) separate linear regressions for each analyte, with analyte concentration as outcome and infection as the predictor variable. Interaction tests were performed to determine if there was an interaction of infection with year on the analyte levels. BH: p-values adjusted for multiple testing by Benjamini-Hochberg¸ CI: Confidence intervals.

|  |  |  |  |  |  |  | **effect on 2010** | |  | **effect on 2013** | |
| --- | --- | --- | --- | --- | --- | --- | --- | --- | --- | --- | --- |
| **analyte** | **coefficient (CI)** | **p-value** | **p-value BH** | **p-value interaction** | **p-value interaction BH** |  | **coefficient (CI)** | **p-value** |  | **coefficient (CI)** | **p-value** |
| IL-1β | 0.895 (0.591, 1.199) | **<0.001** | **<0.001** | **<0.001** | **<0.001** |  | 0.466 (0.148, 0.784) | **0.004** |  | 1.803 (1.536, 2.07) | **<0.001** |
| IL-1RA | -0.076 (-0.165, 0.013) | 0.094 | **0.134** | 0.059 | 0.136 |  | 0.001 (-0.09, 0.091) | 0.988 |  | -0.136 (-0.244, -0.029) | **0.014** |
| TNF | -0.022 (-0.169, 0.124) | 0.768 | **0.818** | 0.895 | 0.926 |  | 0 (-0.146, 0.146) | 0.998 |  | 0.018 (-0.231, 0.267) | 0.887 |
| IL-6 | 0.305 (0.007, 0.603) | **0.045** | **0.068** | **<0.001** | **<0.001** |  | 0.052 (-0.161, 0.264) | 0.632 |  | 0.983 (0.724, 1.241) | **<0.001** |
| IFN-α | 0.011 (-0.036, 0.057) | 0.653 | **0.783** | **<0.001** | **<0.001** |  | -0.085 (-0.133, -0.037) | **0.001** |  | 0.182 (0.107, 0.256) | **<0.001** |
| IFN-γ | 0.229 (0.179, 0.28) | **<0.001** | **<0.001** | 0.479 | 0.684 |  | 0.245 (0.183, 0.306) | **<0.001** |  | 0.207 (0.118, 0.296) | **<0.001** |
| IL-12 | 0.144 (0.101, 0.187) | **<0.001** | **<0.001** | **0.034** | 0.103 |  | 0.111 (0.055, 0.168) | **<0.001** |  | 0.206 (0.142, 0.27) | **<0.001** |
| IL-17 | 0.964 (0.858, 1.07) | **<0.001** | **<0.001** | **0.048** | 0.121 |  | 1.049 (0.922, 1.176) | **<0.001** |  | 0.831 (0.645, 1.016) | **<0.001** |
| IL-10 | 0.227 (0.119, 0.336) | **<0.001** | **<0.001** | 0.133 | 0.249 |  | 0.178 (0.054, 0.302) | **0.005** |  | 0.341 (0.157, 0.525) | **<0.001** |
| IL-13 | -0.186 (-0.277, -0.095) | **<0.001** | **<0.001** | 0.120 | 0.241 |  | -0.233 (-0.314, -0.153) | **<0.001** |  | -0.089 (-0.283, 0.106) | 0.368 |
| IL-4 | 0.011 (-0.064, 0.087) | 0.770 | **0.818** | **0.027** | 0.091 |  | -0.047 (-0.129, 0.036) | 0.265 |  | 0.123 (-0.016, 0.261) | 0.082 |
| IL-5 | 0.165 (0.068, 0.261) | **0.001** | **0.001** | 0.528 | 0.689 |  | 0.189 (0.075, 0.302) | **0.001** |  | 0.125 (-0.052, 0.302) | 0.166 |
| IL-2 | 1.13 (1.005, 1.254) | **<0.001** | **<0.001** | 0.282 | 0.469 |  | 1.183 (1.026, 1.34) | **<0.001** |  | 1.043 (0.836, 1.249) | **<0.001** |
| IL-2R | 0.26 (0.181, 0.34) | **<0.001** | **<0.001** | 0.940 | 0.940 |  | 0.268 (0.186, 0.35) | **<0.001** |  | 0.262 (0.102, 0.422) | **0.002** |
| IL-15 | 1.613 (1.425, 1.801) | **<0.001** | **<0.001** | 0.847 | 0.926 |  | 1.607 (1.364, 1.85) | **<0.001** |  | 1.645 (1.347, 1.943) | **<0.001** |
| IL-7 | -0.776 (-1.037, -0.516) | **<0.001** | **<0.001** | 0.850 | 0.926 |  | -0.759 (-1.06, -0.457) | **<0.001** |  | -0.71 (-1.14, -0.28) | **0.001** |
| Eotaxin | -0.171 (-0.239, -0.102) | **<0.001** | **<0.001** | 0.600 | 0.750 |  | -0.183 (-0.267, -0.098) | **<0.001** |  | -0.145 (-0.262, -0.028) | **0.015** |
| IL-8 | -0.01 (-0.245, 0.226) | 0.937 | **0.937** | **0.019** | 0.070 |  | -0.052 (-0.242, 0.138) | 0.590 |  | 0.275 (0.112, 0.437) | **0.001** |
| IP-10 | -0.434 (-0.514, -0.354) | **<0.001** | **<0.001** | 0.196 | 0.346 |  | -0.46 (-0.559, -0.361) | **<0.001** |  | -0.36 (-0.472, -0.247) | **<0.001** |
| MCP1 | -0.01 (-0.086, 0.066) | 0.790 | **0.818** | **0.000** | **0.002** |  | -0.084 (-0.175, 0.008) | 0.072 |  | 0.154 (0.082, 0.225) | **<0.001** |
| MIG | 0.19 (0.098, 0.282) | **<0.001** | **<0.001** | 0.879 | 0.926 |  | 0.188 (0.062, 0.313) | **0.004** |  | 0.202 (0.072, 0.333) | **0.003** |
| MIP-1α | 0.139 (-0.036, 0.314) | 0.118 | **0.161** | 0.339 | 0.534 |  | 0.142 (-0.047, 0.331) | 0.140 |  | 0.269 (0.136, 0.403) | **<0.001** |
| MIP-1β | 0.077 (-0.101, 0.254) | 0.396 | **0.495** | 0.505 | 0.689 |  | 0.111 (-0.04, 0.262) | 0.149 |  | 0.183 (0.067, 0.298) | **0.002** |
| RANTES | 0.01 (-0.049, 0.069) | 0.741 | **0.818** | 0.822 | 0.926 |  | -0.001 (-0.082, 0.079) | 0.979 |  | 0.012 (-0.063, 0.088) | 0.745 |
| EGF | 0.913 (0.695, 1.132) | **<0.001** | **<0.001** | **<0.001** | **<0.001** |  | 0.598 (0.377, 0.82) | **<0.001** |  | 1.543 (1.229, 1.858) | **<0.001** |
| FGF | 1.782 (1.571, 1.993) | **<0.001** | **<0.001** | **0.010** | **0.044** |  | 2.014 (1.763, 2.265) | **<0.001** |  | 1.461 (1.106, 1.816) | **<0.001** |
| G-CSF | 0.187 (0.124, 0.251) | **<0.001** | **<0.001** | 0.085 | 0.182 |  | 0.152 (0.092, 0.212) | **<0.001** |  | 0.262 (0.135, 0.389) | **<0.001** |
| GM-CSF | 0.054 (-0.062, 0.171) | 0.358 | **0.467** | 0.423 | 0.634 |  | 0.101 (-0.041, 0.244) | 0.162 |  | 0.006 (-0.186, 0.197) | 0.953 |
| HGF | 0.162 (0.096, 0.228) | **<0.001** | **<0.001** | **0.045** | 0.121 |  | 0.122 (0.049, 0.195) | **0.001** |  | 0.251 (0.142, 0.361) | **<0.001** |
| VEGF | 0.667 (0.443, 0.89) | **<0.001** | **<0.001** | **<0.001** | **<0.001** |  | 0.354 (0.151, 0.556) | **0.001** |  | 1.328 (1.051, 1.604) | **<0.001** |

**Table S3. Effect of infection on analyte concentrations and the interactions with year** BH: p-values adjusted for multiple testing by Benjamini-Hochberg.

|  | **Infected** | | **Uninfected** | |
| --- | --- | --- | --- | --- |
| **analytes** | **p-value** | **BH p-value** | **p-value** | **BH p-value** |
| IL-1β | 0.269 | 0.581 | 0.112 | 0.392 |
| IL-1RA | 0.671 | 0.774 | 0.597 | 0.733 |
| TNF | 0.610 | 0.762 | 0.149 | 0.392 |
| IL-6 | 0.560 | 0.730 | **0.031** | 0.187 |
| IFN-α | 0.087 | 0.521 | 0.119 | 0.392 |
| IFN-γ | 0.734 | 0.786 | 0.928 | 0.928 |
| IL-12 | 0.190 | 0.581 | 0.486 | 0.663 |
| IL-17 | 0.399 | 0.638 | 0.320 | 0.524 |
| IL-10 | 0.240 | 0.581 | 0.429 | 0.632 |
| IL-13 | 0.161 | 0.581 | 0.905 | 0.928 |
| IL-4 | 0.271 | 0.581 | 0.329 | 0.524 |
| IL-5 | 0.176 | 0.581 | 0.873 | 0.928 |
| IL-2 | 0.346 | 0.638 | 0.442 | 0.632 |
| IL-2R | 0.253 | 0.581 | 0.541 | 0.706 |
| IL-15 | 0.522 | 0.712 | 0.149 | 0.392 |
| IL-7 | 0.425 | 0.638 | 0.215 | 0.440 |
| Eotaxin | **0.026** | 0.396 | 0.146 | 0.392 |
| IL-8 | 0.253 | 0.581 | 0.157 | 0.392 |
| IP-10 | 0.416 | 0.638 | 0.636 | 0.734 |
| MCP1 | 0.500 | 0.712 | **0.044** | 0.220 |
| MIG | 0.243 | 0.581 | 0.181 | 0.418 |
| MIP-1α | 0.972 | 0.972 | 0.841 | 0.928 |
| MIP-1β | 0.361 | 0.638 | 0.611 | 0.733 |
| RANTES | 0.777 | 0.803 | 0.332 | 0.524 |
| EGF | 0.397 | 0.638 | **0.025** | 0.187 |
| FGF | 0.720 | 0.786 | 0.220 | 0.440 |
| G-CSF | 0.057 | 0.521 | **0.013** | 0.142 |
| GM-CSF | 0.656 | 0.774 | 0.297 | 0.524 |
| HGF | 0.078 | 0.521 | **0.005** | 0.139 |
| VEGF | **0.022** | 0.396 | **0.014** | 0.142 |

**Table S4.** **Effect of sex on analyte concentrations and the interactions with year in infected and uninfected individuals.** The effect of sex (male vs female) on analyte levels was assessed through univariate or multivariable (adjusted models) separate linear regressions for each analyte, with analyte concentration as outcome and sex as the predictor variable. Interaction tests were performed to determine if there was an interaction of sex with year on the analyte levels. BH: p-values adjusted for multiple testing by Benjamini-Hochberg¸ CI: Confidence intervals.

|  | **Infected** | | | | |  | **Uninfected** | | | | |
| --- | --- | --- | --- | --- | --- | --- | --- | --- | --- | --- | --- |
| **analytes** | **coefficient (CI) male vs female** | **p-value** | **p-value BH** | **p-value interaction** | **p-value interaction BH** |  | **coefficient (CI) male vs female** | **p-value** | **p-value BH** | **p-value interaction** | **p-value interaction BH** |
| IL-1β | -0.043 (-0.329, 0.243) | 0.767 | 0.942 | 0.377 | 0.628 |  | -0.309 (-0.805, 0.187) | 0.220 | 0.628 | 0.686 | 0.892 |
| IL-1RA | 0.071 (-0.071, 0.214) | 0.324 | 0.942 | 0.756 | 0.945 |  | 0.085 (-0.03, 0.2) | 0.148 | 0.555 | 0.958 | 0.977 |
| TNF | 0.066 (-0.135, 0.267) | 0.518 | 0.942 | 0.717 | 0.936 |  | -0.079 (-0.291, 0.133) | 0.463 | 0.661 | **0.009** | 0.255 |
| IL-6 | 0.036 (-0.323, 0.395) | 0.842 | 0.950 | 0.372 | 0.628 |  | -0.207 (-0.664, 0.25) | 0.373 | 0.632 | 0.431 | 0.700 |
| IFN-α | -0.002 (-0.069, 0.065) | 0.950 | 0.967 | 0.344 | 0.628 |  | -0.048 (-0.112, 0.017) | 0.146 | 0.555 | 0.075 | 0.323 |
| IFN-γ | 0.037 (-0.025, 0.1) | 0.242 | 0.942 | 0.861 | 0.957 |  | -0.064 (-0.14, 0.012) | 0.101 | 0.555 | **0.045** | 0.323 |
| IL-12 | -0.044 (-0.104, 0.016) | 0.152 | 0.942 | 0.241 | 0.628 |  | 0.015 (-0.048, 0.077) | 0.641 | 0.739 | 0.837 | 0.922 |
| IL-17 | 0.036 (-0.104, 0.177) | 0.612 | 0.942 | 0.336 | 0.628 |  | -0.086 (-0.241, 0.068) | 0.272 | 0.628 | 0.196 | 0.426 |
| IL-10 | -0.015 (-0.18, 0.15) | 0.855 | 0.950 | 0.285 | 0.628 |  | 0.094 (-0.053, 0.24) | 0.210 | 0.628 | 0.604 | 0.892 |
| IL-13 | 0.034 (-0.067, 0.136) | 0.504 | 0.942 | 0.227 | 0.628 |  | -0.063 (-0.206, 0.08) | 0.386 | 0.632 | 0.071 | 0.323 |
| IL-4 | -0.004 (-0.12, 0.113) | 0.952 | 0.967 | 0.314 | 0.628 |  | -0.079 (-0.179, 0.022) | 0.124 | 0.555 | **0.044** | 0.323 |
| IL-5 | 0.063 (-0.084, 0.21) | 0.400 | 0.942 | 0.140 | 0.628 |  | -0.012 (-0.143, 0.119) | 0.858 | 0.858 | 0.053 | 0.323 |
| IL-2 | -0.034 (-0.15, 0.081) | 0.557 | 0.942 | 0.302 | 0.628 |  | -0.083 (-0.287, 0.12) | 0.421 | 0.632 | 0.164 | 0.426 |
| IL-2R | 0.05 (-0.034, 0.135) | 0.242 | 0.942 | 0.129 | 0.628 |  | 0.119 (-0.007, 0.245) | 0.064 | 0.555 | 0.743 | 0.892 |
| IL-15 | -0.003 (-0.124, 0.119) | 0.967 | 0.967 | 0.242 | 0.628 |  | -0.134 (-0.457, 0.189) | 0.415 | 0.632 | 0.356 | 0.628 |
| IL-7 | 0.058 (-0.364, 0.48) | 0.785 | 0.942 | 0.925 | 0.957 |  | -0.303 (-0.637, 0.031) | 0.076 | 0.555 | 0.211 | 0.426 |
| Eotaxin | -0.044 (-0.144, 0.056) | 0.383 | 0.942 | 0.716 | 0.936 |  | -0.045 (-0.14, 0.051) | 0.356 | 0.632 | 0.203 | 0.426 |
| IL-8 | -0.048 (-0.377, 0.281) | 0.774 | 0.942 | 0.543 | 0.814 |  | -0.193 (-0.53, 0.144) | 0.260 | 0.628 | 0.977 | 0.977 |
| IP-10 | 0.036 (-0.094, 0.167) | 0.581 | 0.942 | 0.651 | 0.929 |  | -0.079 (-0.181, 0.024) | 0.131 | 0.555 | 0.861 | 0.922 |
| MCP1 | -0.031 (-0.134, 0.071) | 0.548 | 0.942 | 0.901 | 0.957 |  | -0.027 (-0.139, 0.084) | 0.630 | 0.739 | 0.165 | 0.426 |
| MIG | 0.036 (-0.082, 0.155) | 0.545 | 0.942 | 0.903 | 0.957 |  | -0.124 (-0.26, 0.013) | 0.075 | 0.555 | 0.712 | 0.892 |
| MIP-1α | 0.036 (-0.224, 0.297) | 0.784 | 0.942 | 0.497 | 0.785 |  | -0.047 (-0.289, 0.194) | 0.700 | 0.750 | 0.284 | 0.532 |
| MIP-1β | 0.079 (-0.185, 0.343) | 0.556 | 0.942 | 0.957 | 0.957 |  | -0.085 (-0.329, 0.159) | 0.494 | 0.670 | 0.717 | 0.892 |
| RANTES | 0.06 (-0.029, 0.148) | 0.184 | 0.942 | **0.002** | **0.050** |  | 0.016 (-0.064, 0.096) | 0.695 | 0.750 | 0.141 | 0.426 |
| EGF | -0.099 (-0.289, 0.091) | 0.306 | 0.942 | 0.894 | 0.957 |  | -0.193 (-0.555, 0.168) | 0.293 | 0.629 | 0.818 | 0.922 |
| FGF | 0.067 (-0.238, 0.373) | 0.665 | 0.942 | 0.313 | 0.628 |  | 0.098 (-0.198, 0.394) | 0.514 | 0.670 | 0.058 | 0.323 |
| G-CSF | 0.016 (-0.053, 0.084) | 0.656 | 0.942 | 0.366 | 0.628 |  | 0.03 (-0.07, 0.129) | 0.558 | 0.698 | 0.668 | 0.892 |
| GM-CSF | -0.051 (-0.225, 0.123) | 0.563 | 0.942 | **0.041** | 0.611 |  | 0.024 (-0.136, 0.183) | 0.771 | 0.797 | 0.122 | 0.426 |
| HGF | 0.072 (-0.037, 0.18) | 0.193 | 0.942 | 0.363 | 0.628 |  | -0.049 (-0.132, 0.034) | 0.244 | 0.628 | 0.213 | 0.426 |
| VEGF | 0.073 (-0.127, 0.272) | 0.474 | 0.942 | 0.109 | 0.628 |  | -0.162 (-0.53, 0.206) | 0.387 | 0.632 | 0.443 | 0.700 |

**Table S5. Interaction of neighborhood with year on analyte levels in infected and uninfected individuals.** BH: p-values adjusted for multiple testing by Benjamini-Hochberg.

|  | **Infected** | | **Uninfected** | |
| --- | --- | --- | --- | --- |
| **analytes** | **p-value** | **BH p-value** | **p-value** | **BH p-value** |
| IL-1β | 0.987 | 0.987 | 0.871 | 0.954 |
| IL-1RA | 0.841 | 0.987 | 0.954 | 0.954 |
| TNF | 0.728 | 0.987 | 0.669 | 0.954 |
| IL-6 | 0.795 | 0.987 | 0.866 | 0.954 |
| IFN-α | 0.562 | 0.987 | 0.640 | 0.954 |
| IFN-γ | 0.228 | 0.843 | 0.756 | 0.954 |
| IL-12 | 0.736 | 0.987 | 0.432 | 0.954 |
| IL-17 | 0.284 | 0.853 | 0.304 | 0.954 |
| IL-10 | 0.196 | 0.840 | 0.620 | 0.954 |
| IL-13 | 0.808 | 0.987 | 0.582 | 0.954 |
| IL-4 | 0.725 | 0.987 | 0.912 | 0.954 |
| IL-5 | **0.024** | 0.366 | 0.589 | 0.954 |
| IL-2 | 0.401 | 0.905 | 0.436 | 0.954 |
| IL-2R | 0.253 | 0.843 | 0.229 | 0.954 |
| IL-15 | 0.138 | 0.728 | 0.252 | 0.954 |
| IL-7 | 0.107 | 0.728 | 0.760 | 0.954 |
| Eotaxin | 0.969 | 0.987 | 0.338 | 0.954 |
| IL-8 | 0.379 | 0.905 | 0.614 | 0.954 |
| IP-10 | 0.483 | 0.905 | 0.437 | 0.954 |
| MCP1 | 0.851 | 0.987 | 0.824 | 0.954 |
| MIG | 0.441 | 0.905 | 0.696 | 0.954 |
| MIP-1α | 0.914 | 0.987 | 0.842 | 0.954 |
| MIP-1β | 0.871 | 0.987 | 0.925 | 0.954 |
| RANTES | 0.986 | 0.987 | 0.707 | 0.954 |
| EGF | 0.476 | 0.905 | 0.605 | 0.954 |
| FGF | **0.000** | **0.001** | 0.862 | 0.954 |
| G-CSF | 0.347 | 0.905 | 0.085 | 0.954 |
| GM-CSF | 0.146 | 0.728 | 0.702 | 0.954 |
| HGF | 0.069 | 0.686 | 0.682 | 0.954 |
| VEGF | 0.846 | 0.987 | 0.540 | 0.954 |
