# Supplementary figures and images for: Changing plasma cytokine, chemokine and growth factor profiles upon differing malaria transmission intensities
Source: Malar J. 2019 Dec 5;18:406. doi: 10.1186/s12936-019-3038-x (PMC6896751; doi:10.1186/s12936-019-3038-x)

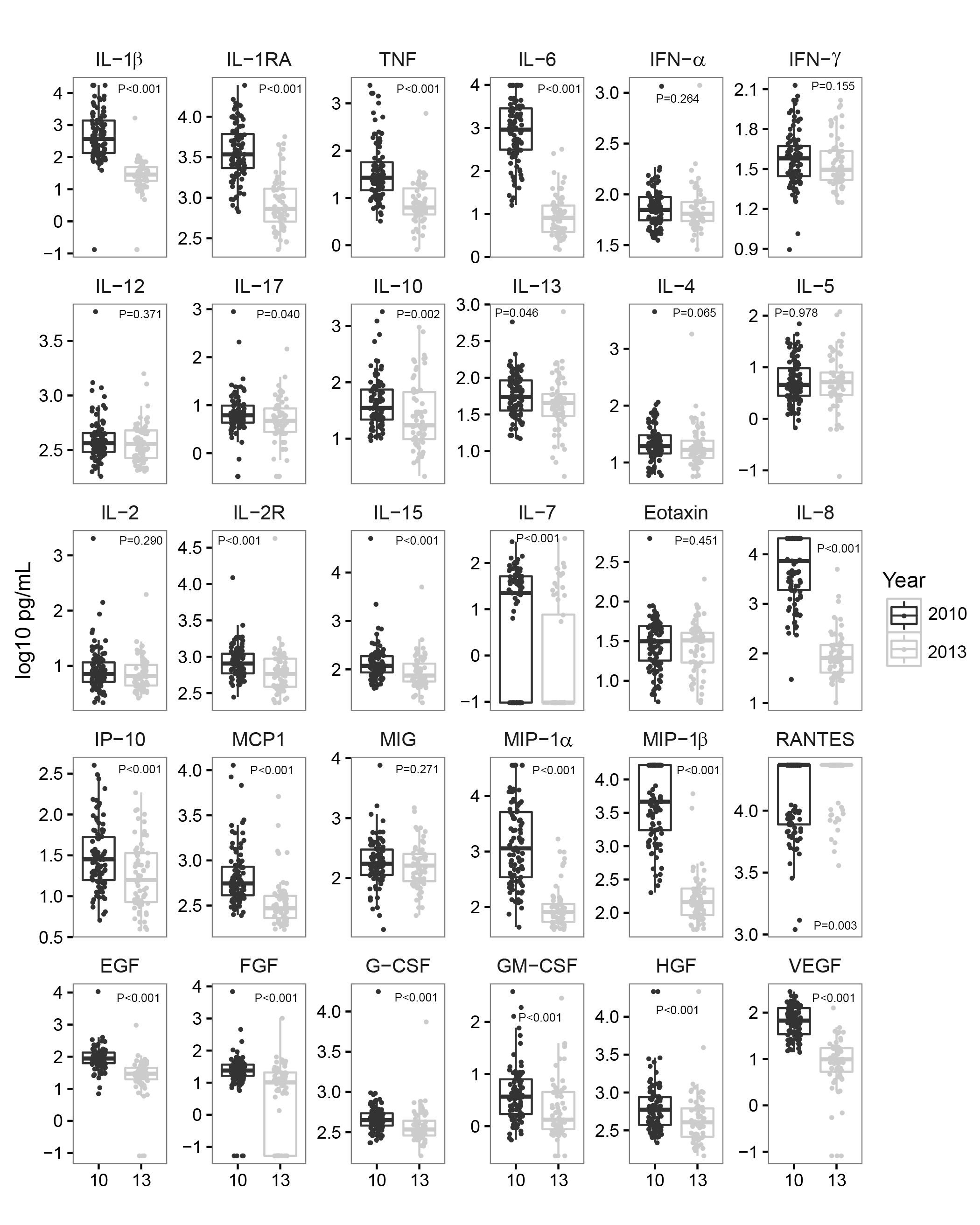

Supplement: Supplementary file 2 — Additional file 2. Differences in cellular immune mediator concentrations between 2010 (lower MTI) and 2013 (higher MTI) in P. falciparum-infected subjects. Box plots representing the median and interquartile range of each analyte concentration (log10 pg/mL) in infected subjects recruited in 2010 and in 2013. Levels between both years were compared by Wilcoxon rank-sum test and p-values were adjusted for multiple testing by Benjamini–Hochberg approach. [file 12936_2019_3038_MOESM2_ESM.tif]

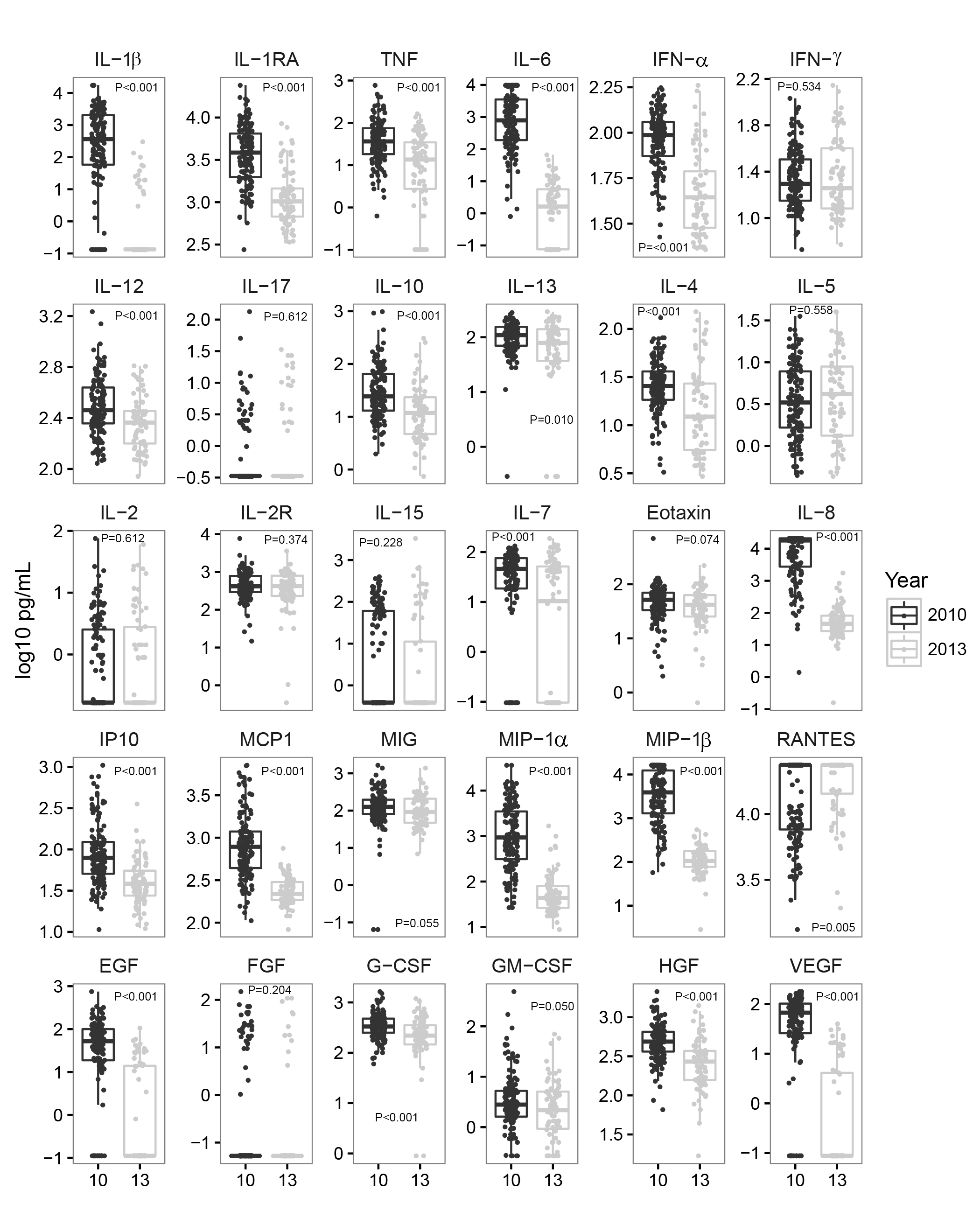

Supplement: Supplementary file 3 — Additional file 3. Differences in cellular immune mediator concentrations between 2010 (lower MTI) and 2013 (higher MTI) in uninfected subjects. Box plots representing the median and interquartile range of each analyte concentration (log10 pg/mL) in uninfected subjects recruited in 2010 and in 2013. Levels between both years were compared by Wilcoxon rank-sum test and p-values were adjusted for multiple testing by the Benjamini–Hochberg approach. [file 12936_2019_3038_MOESM3_ESM.tif]

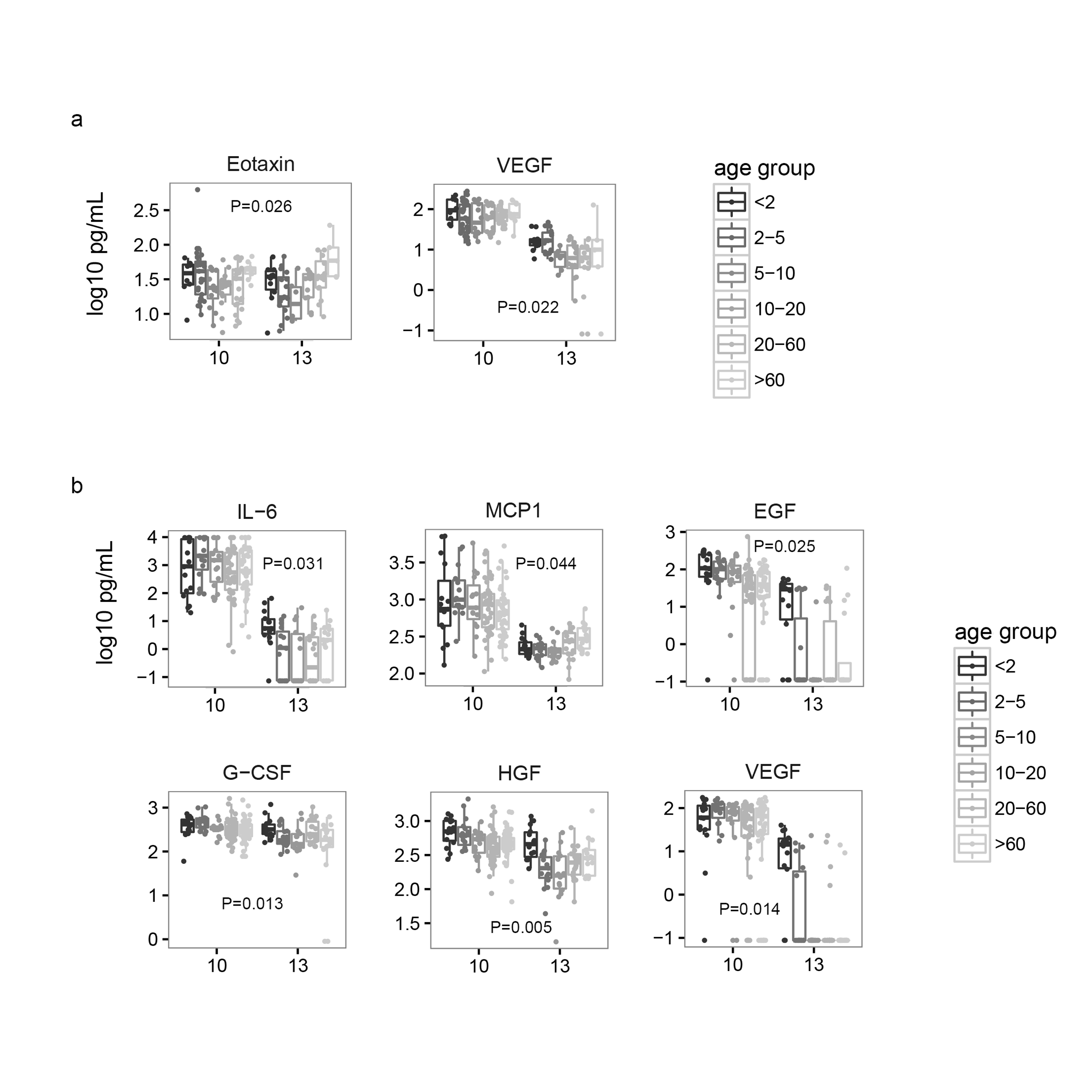

Supplement: Supplementary file 5 — Additional file 5. Cellular immune mediator concentrations in 2010 (low MTI) and 2013 (high MTI) stratified by age group. Box plots representing the median and interquartile range of analytes concentrations (log10 pg/mL) in P. falciparum infected (a) and uninfected (b) subjects. Only analytes in which age and year had a significant p-value for the interaction test (before correcting for multiple testing) are shown. [file 12936_2019_3038_MOESM5_ESM.tif]

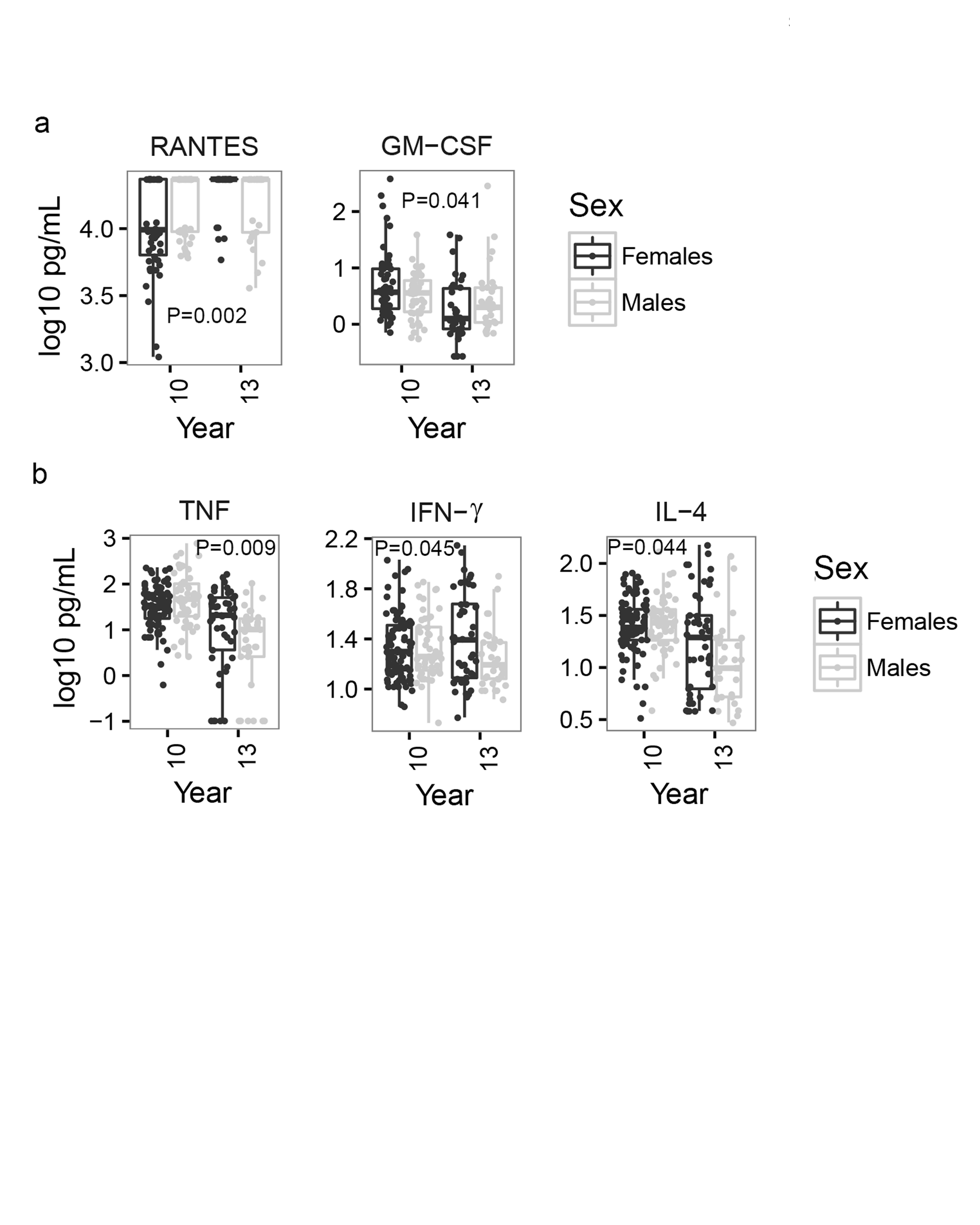

Supplement: Supplementary file 6 — Additional file 6. Cellular immune mediator concentrations in 2010 (low MTI) and 2013 (high MTI) stratified by sex. Box plots representing the median and interquartile range of analyte concentrations (log10 pg/mL) in P. falciparum infected (a) and uninfected (b) subjects. Only analytes in which sex and year had a significant p-value for the interaction test (before correcting for multiple testing) are shown. [file 12936_2019_3038_MOESM6_ESM.tif]

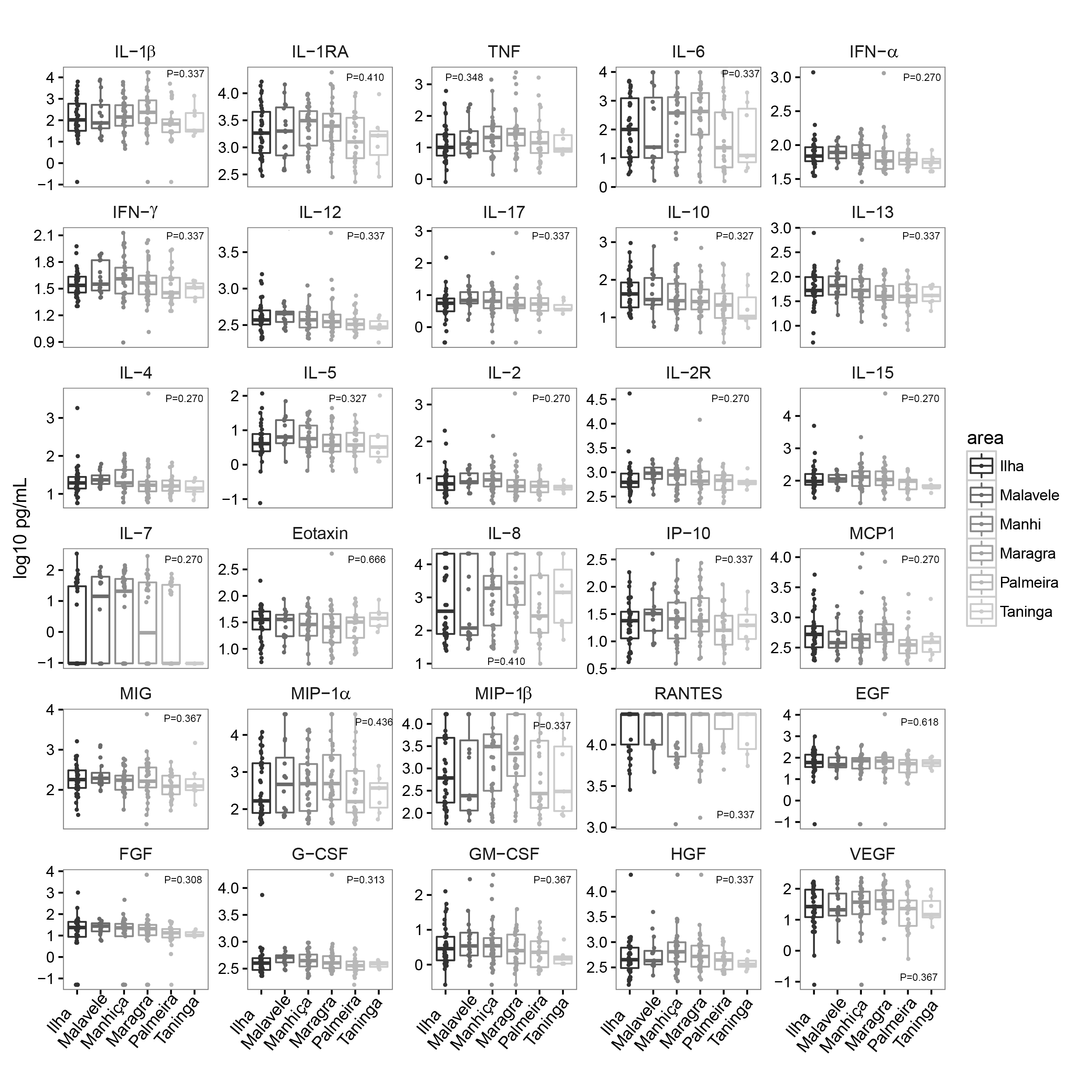

Supplement: Supplementary file 7 — Additional file 7. Differences in cellular immune mediator concentrations between areas in P. falciparum infected subjects. Box plots representing the median and interquartile range of each analyte concentration (log10 pg/mL) in infected subjects stratified by neighborhood. Levels between areas have been compared by Kruskal–Wallis test. [file 12936_2019_3038_MOESM7_ESM.tif]

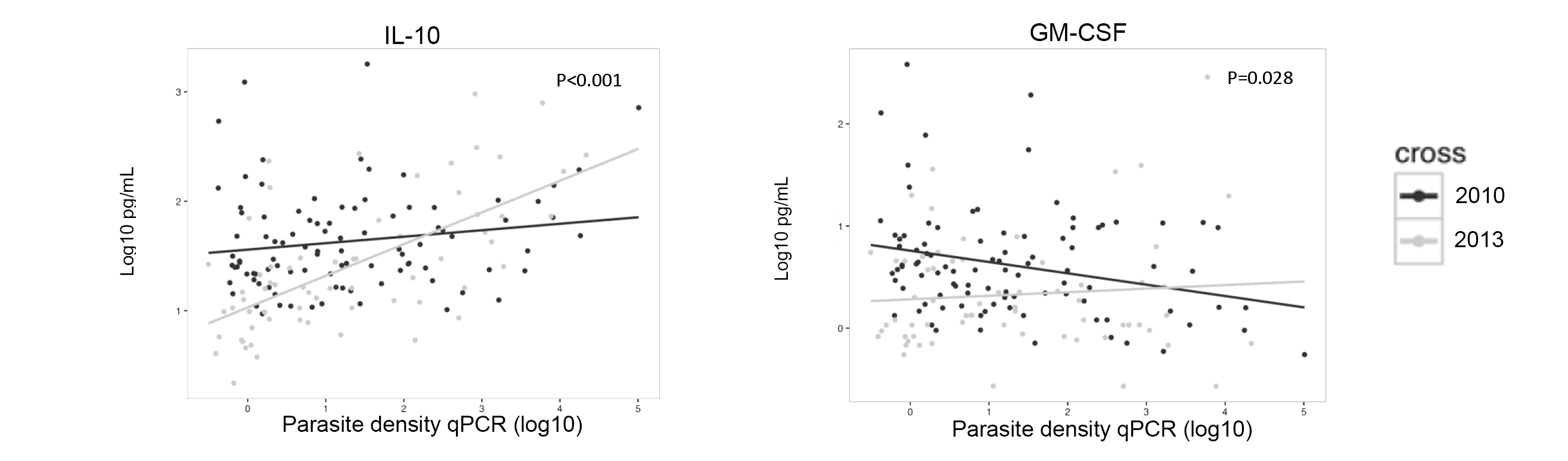

Supplement: Supplementary file 8 — Additional file 8. Effect of parasitema on IL-10 and GM-CSF concentrations stratified by year. Scatter plots with trend line representing the distribution of analytes concentration by parasitemia stratified by year. Only analytes in which parasitemia and year had a significant p-value for the interaction test (before correcting for multiple testing) are shown. [file 12936_2019_3038_MOESM8_ESM.tif]
