# Supplementary material for: Changing plasma cytokine, chemokine and growth factor profiles upon differing malaria transmission intensities
Source: Malar J. 2019 Dec 5;18:406. doi: 10.1186/s12936-019-3038-x (PMC6896751; doi:10.1186/s12936-019-3038-x)
Supplement: Supplementary file 4 — Additional file 4. Correlations of VEGF, MCP-1 and IFN-α with P. falciparum antibody levels stratified by year. Data are presented in scatter plots with trend lines, with analytes concentrations in the Y axes and antibody levels in the X axes. R2 and p-values were obtained through linear regression models. Only analytes that had a statistically significant interaction with year for the correlation with antibodies are shown. [file 12936_2019_3038_MOESM4_ESM.pdf]

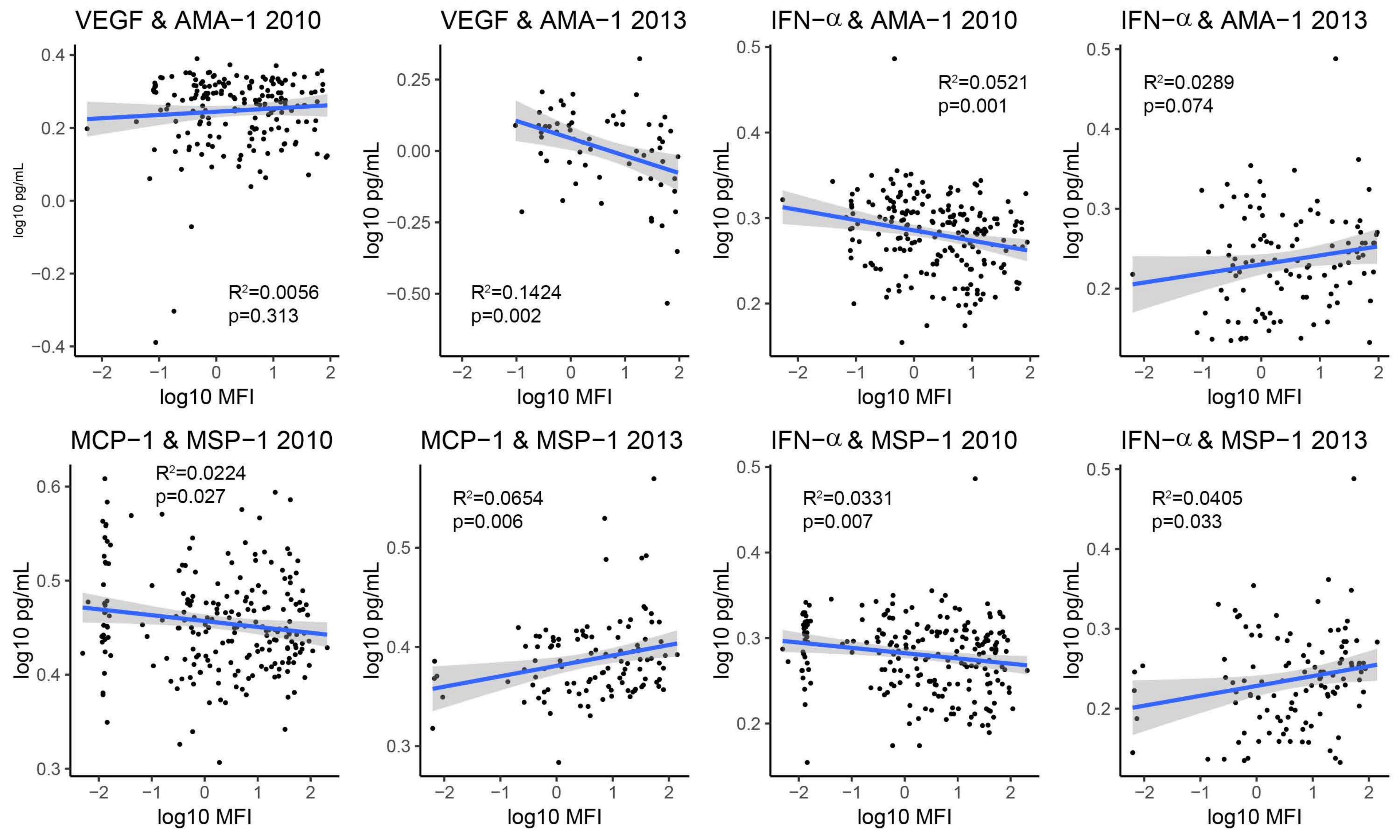
VEGF & AMA−1 2010
log10 pg/mL
log10 MFI
R²=0.0056
p=0.313
VEGF & AMA−1 2013
log10 pg/mL
log10 MFI
R²=0.1424
p=0.002
IFN−α & AMA−1 2010
log10 pg/mL
log10 MFI
R²=0.0521
p=0.001
IFN−α & AMA−1 2013
log10 pg/mL
log10 MFI
R²=0.0289
p=0.074
MCP−1 & MSP−1 2010
log10 pg/mL
log10 MFI
R²=0.0224
p=0.027
MCP−1 & MSP−1 2013
log10 pg/mL
log10 MFI
R²=0.0654
p=0.006
IFN−α & MSP−1 2010
log10 pg/mL
log10 MFI
R²=0.0331
p=0.007
IFN−α & MSP−1 2013
log10 pg/mL
log10 MFI
R²=0.0405
p=0.033
